# Characterization of microRNAs Expressed during Secondary Wall Biosynthesis in *Acacia mangium*

**DOI:** 10.1371/journal.pone.0049662

**Published:** 2012-11-27

**Authors:** Seong Siang Ong, Ratnam Wickneswari

**Affiliations:** School of Environmental and Natural Resource Sciences, Faculty of Science and Technology, Universiti Kebangsaan Malaysia, Bangi, Selangor, Malaysia; Lawrence Berkeley National Laboratory, United States of America

## Abstract

MicroRNAs (miRNAs) play critical regulatory roles by acting as sequence specific guide during secondary wall formation in woody and non-woody species. Although thousands of plant miRNAs have been sequenced, there is no comprehensive view of miRNA mediated gene regulatory network to provide profound biological insights into the regulation of xylem development. Herein, we report the involvement of six highly conserved amg-miRNA families (amg-miR166, amg-miR172, amg-miR168, amg-miR159, amg-miR394, and amg-miR156) as the potential regulatory sequences of secondary cell wall biosynthesis. Within this highly conserved amg-miRNA family, only amg-miR166 exhibited strong differences in expression between phloem and xylem tissue. The functional characterization of amg-miR166 targets in various tissues revealed three groups of HD-ZIP III: ATHB8, ATHB15, and REVOLUTA which play pivotal roles in xylem development. Although these three groups vary in their functions, -psRNA target analysis indicated that miRNA target sequences of the nine different members of HD-ZIP III are always conserved. We found that precursor structures of amg-miR166 undergo exhaustive sequence variation even within members of the same family. Gene expression analysis showed three key lignin pathway genes: *C4H*, *CAD*, and *CCoAOMT* were upregulated in compression wood where a cascade of miRNAs was downregulated. This study offers a comprehensive analysis on the involvement of highly conserved miRNAs implicated in the secondary wall formation of woody plants.

## Introduction

Recently, tremendous interest has been devoted to better understand the molecular basis of wood formation. Wood formation involves several events, from the differentiation and division of the xylem cells until the formation of heartwood tissues [1]. Elucidation of the mechanisms involved in producing secondary walls will help plant biologists to optimize the production of environmentally cost-effective and renewable alternative resources as wood has been widely used for pulping, timber industry and biofuel industry [2]. In the last decades, genomics studies in model species have revealed various wood associated genes playing an independent and additive role during secondary wall formation [3], [4].

Genomics studies in herbaceous and woody species have identified pathway genes playing an interconnected role in lignin biosynthesis. These complexities reveal groups of transcriptional factors playing an independent and additive regulatory role in lignin biosynthesis [5]. Although important progress has been made into the identification of transcriptional factors that bind the AC elements of monolignol biosynthetic pathway genes, little is known about the transcriptional regulators that are responsible for turning on the secondary wall biosynthetic program [2]. This was hampered by the overlapping roles in the transcriptional regulation of lignin biosynthetic pathway with the biosynthesis of other secondary wall components [6]. Such a multilayered regulatory network affects multiple target genes and also points to the existence of switches that can be tuned to regulate different cell wall pathways in new spatial and temporal patterns [7].

In model plant species, studies have validated the roles of miRNAs in regulating lignin biosynthetic pathway genes. Transcriptional regulation alone is insufficient to provide an efficient fine-tuning of target gene expression in certain cells or tissues. Several studies have suggested that miRNAs play important roles in plant growth and development. More than 21,643 miRNAs have been identified from 168 species (miRBase release 18, November 2011; http://microrna.sanger.ac.uk; http://cgrb.orst.edu/smallRNA/db/). This large numbers of miRNAs identified using cloning, direct sequencing and computational prediction suggest that non-coding RNA play a prominent role in the regulatory networks of plant and animal development. Identification of a cascade of highly conserved miRNA across plant species suggests a common mechanism regulating vascular development in all plants [8]. Small RNAs like miRNAs act as post-transcriptional regulators by regulating the meristem cell differentiation and tissue patterning and have been shown to spatially regulate gene expression at various developmental stages in plants [9], [10].

Deep sequencing of small RNAs has suggested that certain miRNA families are indeed conserved across diverse plant species. Although important progress has been made in characterizing all miRNAs in plants, limited information to justify their roles in various tissues and developmental stages has hampered our understanding of their roles. miRNAs have been reported to play important roles in plant growth and development [11]. In this study, we detected the expression of highly conserved amg-miRNAs in various wood forming tissues across different genotypes in *A. mangium*. Expression patterns of the highly conserved amg-miRNAs in different tissues suggested amg-miR166 as the potential regulatory sequence in vascular tissues. Subsequently, mapping of the amg-miR166 cleavage site via 5′RACE and 3′RACE were employed to isolate the gene targets. Bioinformatics pipeline analysis and expression studies in high and low lignin tissues were undertaken to unravel the regulatory roles of this amg-miR166. Our study also suggests miRNAs may play an important regulatory role in monolignol biosynthetic pathways despite the complexity of miRNA interaction in secondary wall formation.

## Results

### Expression profile of highly conserved miRNA families in various tissues in *A. mangium*

Despite the importance of small RNAs in plant growth and development, information on the roles of small RNA in *Acacia mangium* is still in its infancy. Unlike *Arabidopsis* and *poplar*, potential roles of highly conserved miRNA families in various wood forming tissues in *A. mangium* are unclear. From the expression profiles of small RNAs generated between low lignin Am54 and high lignin Am48 [12], we have identified six highly conserved miRNA families that are potentially important regulatory sequences in secondary wall formation (Table S1). We report here the expression level of these six miRNAs in various wood forming tissues across different genotypes of four years *A. mangium* using quantitative real time - PCR.

**Figure 1:**
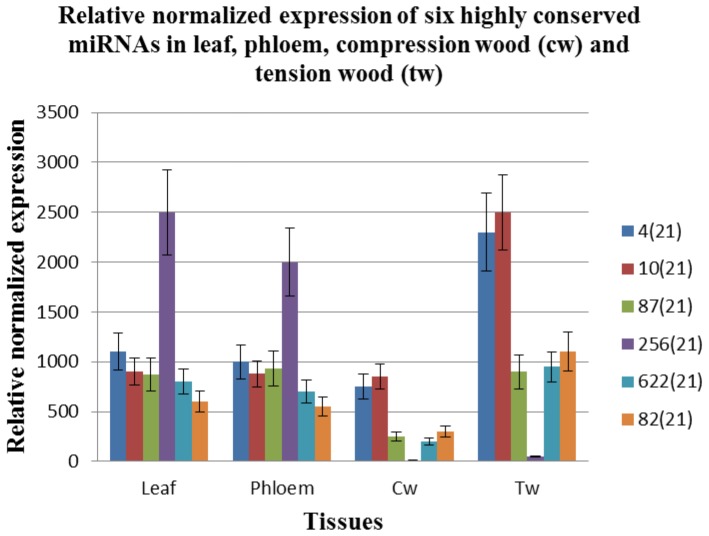
RT-qPCR analysis of six microRNA members in various tissues.

Altogether, we have identified six amg-miRNA families with potentially important regulatory roles in secondary wall biosynthesis from their expression profile in high lignin compression wood and low lignin tension wood across three different genotypes (Figure 1). Secondary cell walls are composed of cellulose, hemicellulose and lignin. This novel discovery is consistent with the findings reported earlier across three 2-year-old *A. mangium* genotypes: NSW22, AVW22 and WMH16 [12]. Hence, these highly conserved amg-miRNA families may be important regulators in secondary wall formation since genotype and age differences have minimal impacts on their expression patterns.

Our findings suggest the involvement of a well conserved regulatory network in secondary wall forming cells with some miRNA families exhibiting dual roles. This is evident as amg-miR172, amg-miR156, amg-miR168, amg-miR159 and amg-miR394 exhibited more or less similar expression between phloem and xylem wood tissues (Figure 1). Although the five amg-miRNAs showed differences between compression and tension wood (Figure 1), the presence of these amg-miRNA in phloem tissues indicates their potential involvement in phloem and xylem development. Only amg-miR166 exhibited strong downregulation in xylem tissues which suggests its roles in xylem formation and lignin regulation for further validation.

### Identification of key regulatory sequences in vascular tissue development through quantitative real time-PCR

To improve our understanding on the complexity in the regulatory network in vascular tissues, we used quantitative real-time PCR technology to track the changes in miRNA expression in various wood forming tissues. We selected one miRNA with highest expression among the members in each family from six different families (Table S1) and examined their expression in leaf, phloem, compression wood and tension wood. We compared the ct value of the selected miRNA member from each family.

Of the six different miRNA members, we found that amg-miR166 has the highest ct value in compression wood and tension wood (Table 1). Ct values are inversely proportional to the amount of target nucleic acid in a sample. Every increase in 3.33 ct value represented a tenfold decrease in concentration of the investigated target nucleic acid. Phloem tissues showed a -1000 increase in amg-miR166 concentration as compared to xylem tissues (Figure 1) implying the strong downregulation of this miRNA from phloem to xylem tissue. Moreover, near absence of this miRNA in compression wood and tension wood as compared to other amg-miRNAs using quantitative real time PCR requires in-depth studies to verify the role of this miRNA in xylem formation and lignin regulation in *A. mangium*. This expression pattern is similar to previous studies reporting miR166 playing an important role in vascular development and lignin regulation [8], [12], [13], [14], [15], [16], [17].

**Table 1:** Mean cycle threshold (ct) value of different miRNA members in compression wood (cw) and tension wood (tw) of (A) two years old and (B) four years old *A. mangium* trees with three biological replicates for each tree.

(A)			
Sequence ID	amg-miRNA families (genotypes)	Cw (mean ct value)	Tw (mean ct value)
4(21)	amg-miR168 (NSW22)	22.30	20.62
256(21)	amg-miR166(NSW22)	30.04	28.62
10(21)	amg-miR172(NSW22)	22.32	20.74
87(21)	amg-miR159(NSW22)	22.28	20.63
622(21)	amg-miR394(NSW22)	24.88	21.94
82(21)	amg-miR156(NSW22)	23.56	21.94
4(21)	amg-miR168 (AVW22)	23.23	21.37
256(21)	amg-miR166(AVW22)	30.55	28.92
10(21)	amg-miR172(AVW22)	22.84	21.27
87(21)	amg-miR159(AVW22)	25.13	23.56
622(21)	amg-miR394(AVW22)	25.66	22.50
82(21)	amg-miR156(AVW22)	24.44	23.01
4(21)	amg-miR168(WMH16)	24.12	22.32
256(21)	amg-miR166(WMH16)	33.52	31.56
10(21)	amg-miR172(WMH16)	18.66	17.12
87(21)	amg-miR159(WMH16)	28.13	26.46
622(21)	amg-miR394(WMH16)	26.81	23.59
82(21)	amg-miR156(WMH16)	23.13	21.66

(B)			
Sequence ID	amg-miRNA families (genotypes)	Cw (mean ct value)	Tw (mean ct value)
4(21)	amg-miR168 (NSW19)	19.72	17.93
256(21)	amg-miR166(NSW19)	37.54	35.74
10(21)	amg-miR172(NSW19)	18.66	17.22
87(21)	amg-miR159(NSW19)	23.20	21.80
622(21)	amg-miR394(NSW19)	32.67	29.71
82(21)	amg-miR156(NSW19)	22.34	20.67
4(21)	amg-miR168 (NSW20)	22.16	20.18
256(21)	amg-miR166(NSW20)	32.78	30.95
10(21)	amg-miR172(NSW20)	19.29	17.84
87(21)	amg-miR159(NSW20)	24.78	22.22
622(21)	amg-miR394(NSW20)	24.12	21.15
82(21)	amg-miR156(NSW20)	23.56	22.00
4(21)	amg-miR168(ERC22)	21.64	19.87
256(21)	amg-miR166(ERC22)	33.28	31.89
10(21)	amg-miR172(ERC22)	20.40	18.88
87(21)	amg-miR159(ERC22)	21.10	19.58
622(21)	amg-miR394(ERC22)	22.34	19.63
82(21)	amg-miR156(ERC22)	21.96	20.13

Ct value of the 5.8 S ribosomal RNA in compression wood and tension wood across investigated genotypes were fixed at 9.00.

### Experimental and binding energy characterization of genes regulated by miR166

**Figure 2.Mapping:**
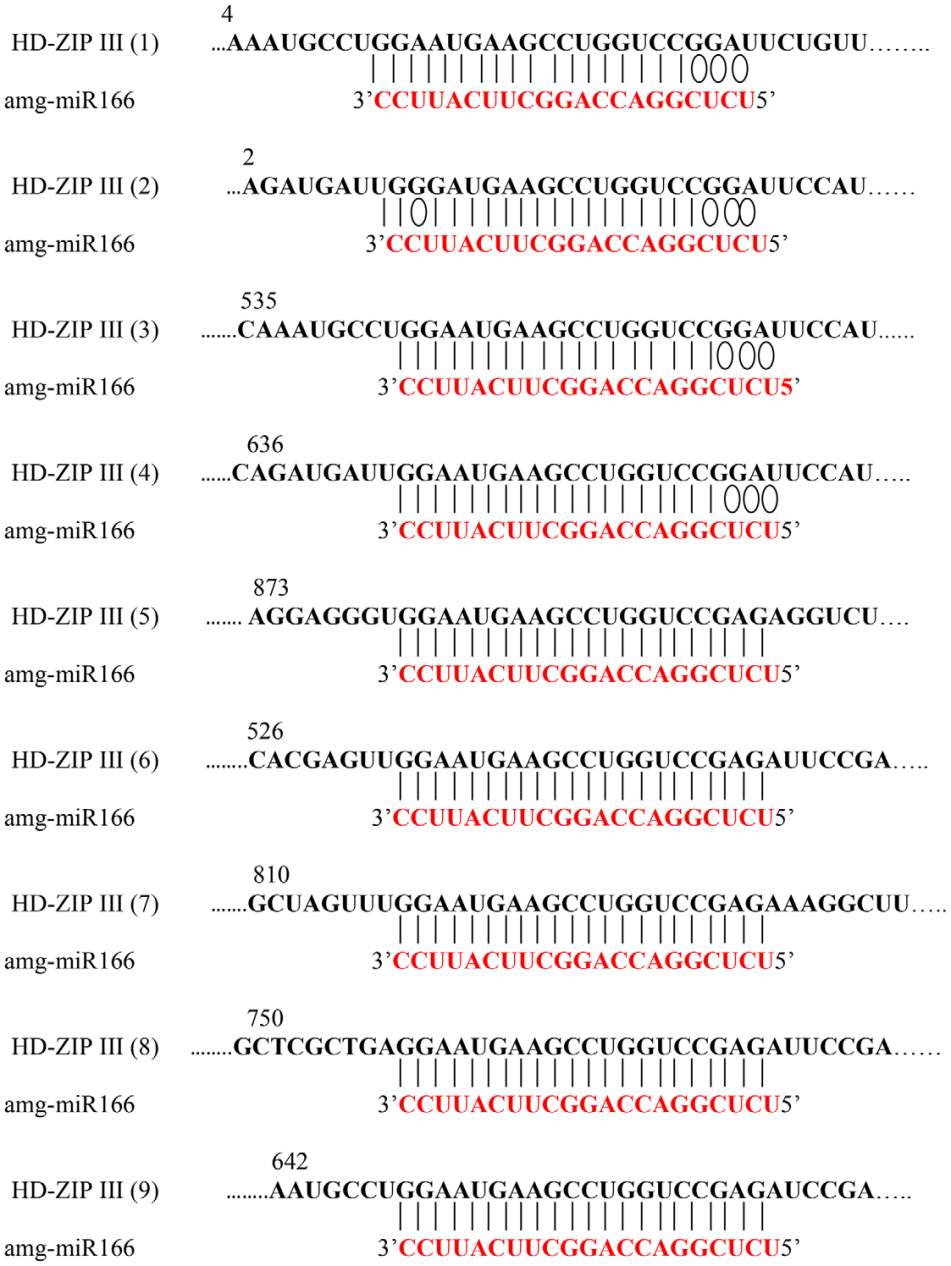
of the amg-miR166 cleavage sites by 5′ Rapid Amplification of cDNA Ends (RACE).

**Figure 3:**
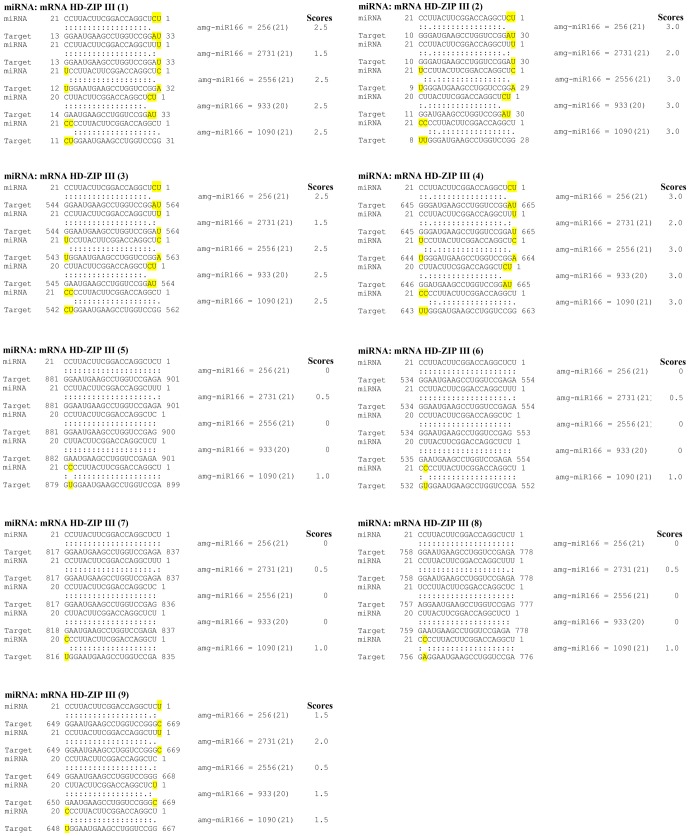
Binding energy characterization of nine HD-ZIP III transcripts with various isoforms of amg-miR166.

To strengthen our findings and to understand how miRNAs regulate their target genes [11], [18], [19], we performed 3′- and 5′- RACE using various tissues. Pooled xylem tissues (from compression wood and tension wood) were used to obtain different members of HD-ZIP III regulated by amg-miR166. We were unable to obtain any HD-ZIP III members via 5′RACE mapping of amg-miR166 cleavage site in leaf and phloem tissues. RACE PCR products revealed nine mRNA are indeed the gene target of amg-miR166 (Figure 2). All the amg-miR166 binding site are located at the 5′UTR of the characterized HD-ZIP III mRNA. Using psRNA Target Analysis, we found that all our HD-ZIP III mRNA will be cleaved by amg-miR166 with scores of 3.0 and below regardless of the polymorphism in the sequences (Figure 3).

5′ RACE mapping of miRNA cleavage site revealed nine different members of HD-ZIP III transcription factors were successfully characterized in this study. We obtained five novels HD-ZIP III mRNA out of the nine members (Figure S1). HD-ZIP III (1) shared 91% in nucleotide level and 89% in protein level to *Glycine max* HD-ZIP III (REVOLUTA) transcription factors with e-value of 0. HD-ZIP III (2) shared 92% in nucleotide and protein level to *Medicago truncatula* HD-ZIP III (ATHB15 group) transcription factors with e-value of 0. HD-ZIP III (3) shared 91% in nucleotide and 92% in protein level to *Medicago truncatula* HD-ZIP III (ATHB15 group). HD-ZIP III (4) shared 89% in nucleotide and 88% in protein level to *Populus trichocarpa* class HD-ZIP III (ATHB8 group). Blast analysis at nucleotide level between HD-ZIP 2 and HD-ZIP 3 revealed that both of them share 87% similarity in nucleotide and 92% in protein level (data not shown). Although *Arabidopsis* genome contains five class III HD-ZIP genes (ATHB8, PHAVOLUTA/ATHB9, PHABULOSA/ATHB14, CORONA/ATHB15 and REVOLUTA/IFL1) which are targeted by miR166, our study revealed only three different class III HD-ZIP are present in *A. mangium*. In summary, blast analysis at the nucleotide and protein level indicated all the isolated HD-ZIP III transcription factors are different candidate genes.

### Real-time PCR validation of HD-ZIP III transcription factors

To pinpoint the role of each HD-ZIP III transcription factor in wood forming tissues, we analyzed their expression level in various wood forming tissues. Preferential expressions in each of the different transcription factors in various tissues suggest a potential role in xylem formation. Interestingly, the four different HD-ZIP III transcription factors were abundantly expressed in compression wood and their expression was downregulated in tension wood, tissue characterized by lower lignin content. They were weakly expressed in phloem tissues and absent in leaf tissues.

**Figure 4:**
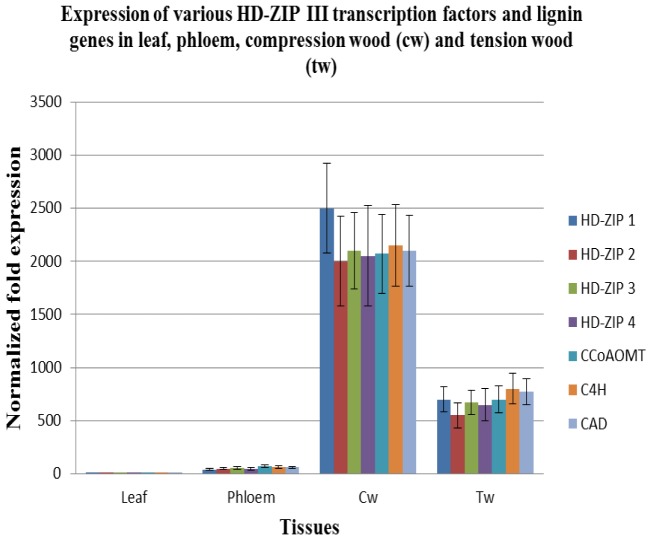
RT-qPCR of transcription factors and key lignin genes.

From the expression data of these four different HD-ZIP III as presented in Figure 4, we suggest that amg-miR166 play a pivotal role in maintaining the abundancy of the various members of HD-ZIP III transcripts through their miRNA cleavage site and also via epigenetic silencing. Hence, the study presented here strongly suggests vascular tissue development is very complex and require the involvement of various members from a particular family (Figure 3) which are in agreement with other studies [8], [9], [15].

### Sequence comparison of conserved and novel miRNA target

**Figure 5:**
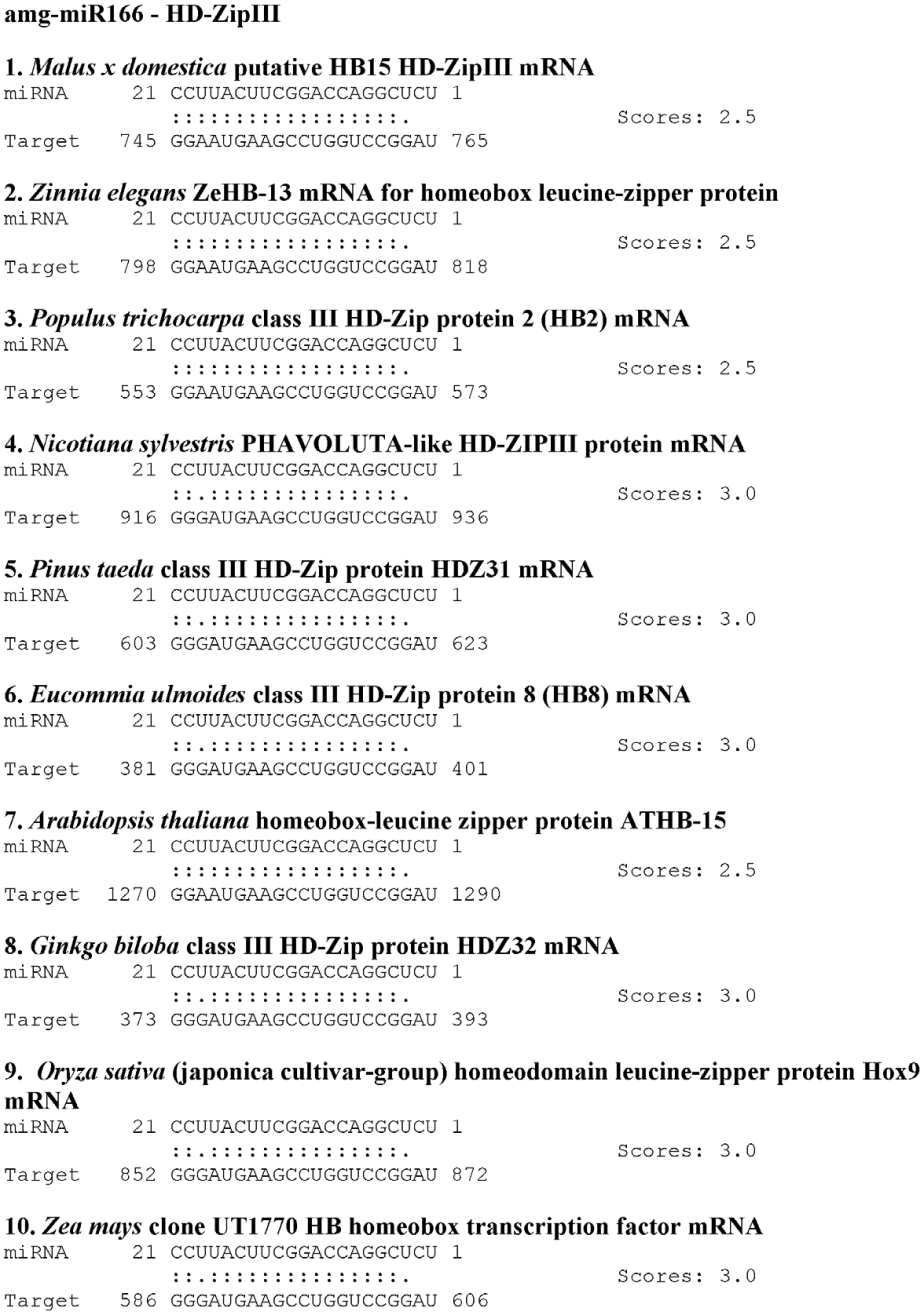
Binding energy characterization between amg-miR166 with mRNA from various plant species.

As both miRNAs and their corresponding target site are highly conserved across a wide range of species [20], information on the homologous miRNA and mRNA dataset will provide useful information on conserved regulatory roles even in different species [21]. To prove whether the homologous miRNA and mRNA in other species have a similar sequence conservation and binding energy with our species, we isolated the mRNA target of amg-miR166 using 5′RACE and 3′RACE approach. We chose amg-miR166 in this study because this miRNA showed the greatest difference in its expression between phloem and xylem tissue compared to the other miRNAs. Moreover, it had the lowest abundance in compression wood and tension wood among the various amg-miRNA families chosen for validation in this study. This amg-miR166 is the first plant miRNA identified and is found in all plant species. Bioinformatics analysis using psRNA Target Analysis Server between amg-miR166 with HD-ZIP III from ten different species indicated that variations in the miRNA: mRNA target sequences only occur at the 3′ end of miRNA (Figure 5). We verified here the four isolated HD-ZIP III mRNA (HD-ZIP III (1–4) share high similarity with other species at nucleotide and protein levels (Figure S1). In addition to these four different HD-ZIP III mRNA, we also obtained five novel amg-miR166 targets cleavage site (HD-ZIP III (5–9) (Figure S1).

### Expression of *C4H*, *CCoAOMT* and *CAD* genes in high lignin compression wood and low lignin tension wood

Previous investigation has identified six genes involved in lignin biosynthetic pathway in *Acacia mangium* x *Acacia auriculiformis* hybrid: caffeate O-methyltransferase (*COMT*), cinnamoyl-coenzyme A reductase (*CCR*), cinnamyl-alcohol dehydrogenase (*CAD*), phenylalanine ammonia-lyase (*PAL*), cinnamate 4-hydroxylase (*C4H*) and caffeoyl-coenzyme A O-methytransferase (*CCoAOMT*) [22]. *C4H*, *CCoAOMT* and *CAD* are three enzymes that play an important role in the beginning, middle and the end of the lignin biosynthetic pathway [3]. *C4H*, *CCoAOMT* and *CAD* have been isolated and characterized in *A. mangium* x *A. auriculiformis* hybrid [23]. In this study, downregulation of these six highly conserved miRNAs in high lignin compression wood as presented in Fig. 1 was accompanied by the upregulation of the *C4H*, *CCoAOMT* and *CAD* genes (Figure 4). Generally lignin content in compression wood in the different *A. mangium* trees was higher than in tension wood (Table 2). As the expression of major pathway genes involved in secondary wall biosynthesis are highly coordinated and could be switched on and off [16], [24], [25], [26], [27], [28], [29], [30], [31], [32], [33], [34], upregulation and downregulation of miRNA affects not only lignin content but also cellulose and/or hemicellulose levels.

**Table 2:** Lignin content in compression wood and tension wood in four years old *A. mangium* with various genotypes (NSW19, NSW20 and ERC22).

*A. mangium*	Compression wood (%)	Tension wood (%)	Percentage difference (%)
NSW19	32.48	23.8	8.68
NSW20	26.2	17.63	8.49
ERC22	27.3	16.85	10.46

### Characterization of amg-miR166 Precursors

**Figure 6:**
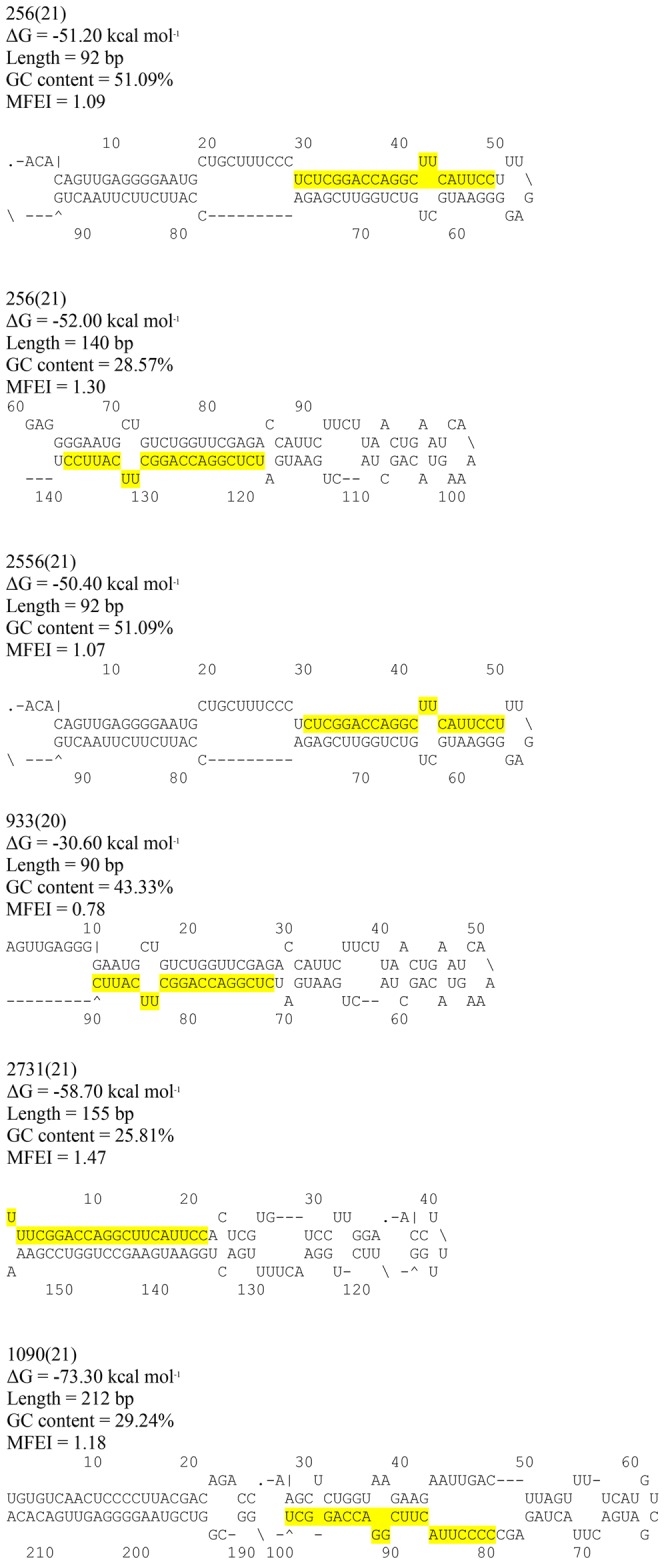
Secondary structure of the five newly cloned amg-miR166 precursors.

Although miR166 has been well studied in various species, no studies have explored the biogenesis of this miRNA in *A. mangium*. Biogenesis of miRNA involves several enzymes and processes [35], [36], [37], [38], [39]. In order to address this question, we first obtained the precursor structure of all the isoforms belonging to amg-miR166 family (Figure 6). Interestingly, the precursor structures varied greatly among all the members of this family. Jones-Rhoades and Bartel [40] reported that miRNA: miRNA* portion of the pre-miRNAs are more conserved than other parts of the precursor's structure even within the same miRNA family. In agreement with Jones-Rhoades and Bartel [40], four out of the five amg-miR166 precursors' structures in this study shared more conserved portion in their miRNA: mRNA* structure.

Several studies in plant genome have proven that similar mature miRNA may arise from multiple precursors. As shown in miRBase, certain isoforms of mature miR166 in *Arabidopsis* and *Poplar* were encoded by more than five different precursor structures. Since *Acacia* genome is not available yet, we are unable to determine the complete precursor structure of this amg-miR166 family. As structure of miRNA precursors may influence the rate of miRNA production [40], identification of the complete set of all amg-miR166 precursor structures is important for the selection of the right amiRNA precursor that can best express mature miRNA. The precursor structure of amg-miR166 obtained in this study fulfilled several parameters; have negative folding free energies [41], MFEI threshold above 0.7 [42], predicted mature miRNA sequence differed from their homologous miRNA by only (0–3) mismatches [42], no large break or loop in the miRNA sequence [42] and range from 90 nucleotides to more than 210 nucleotides (Figure 6). The precursor structure of the different amg-miR166 members obtained can be used for the development of artificial miRNA (amiRNA) gene construct for silencing of HD-ZIP III transcription factors.

## Discussion

### Regulation of class III HD-ZIP transcription factor via post-transcriptional and transcriptional gene silencing

The first plant genes identified to be the target under miRNA regulation are HD-ZIP III transcription factors [43]. Bioinformatics analysis using psRNA server indicated all the five different members belonging to the amg-miR166 family in our study will bind to the nine different HD-ZIP III transcripts and direct its cleavage (Figure 3). We demonstrate that most of HD-ZIP III transcripts in compression wood and tension wood are safe from low abundance of amg-miR166 cleavage. No direct evidence of HD-ZIP III transcripts cleavage by amg-miR166 in leaf and phloem tissues at the expected site was found. However, the presence of high level of CpG island in the HD-ZIP III transcripts (Figure S1) could contribute to high level of methylation which have been associated with low rates of transcription of miR166 targets [44]. Hence, amg-miR166 was suggested to play a critical regulatory role via RNAi and epigenetic silencing in controlling gene expression in secondary wall formation in *A. mangium*. We show that expression of amg-miR166 was corroborated with the expression of HD-ZIP III transcripts in this study (Figure 1 and 4). For instance, upregulation of miR166 in leaf was accompanied with strong downregulation of their transcripts and similar expression also reported in phloem, compression wood and tension wood in this study.

HD-ZIP III transcription factors play an important role in xylem development. Overexpression of ATHB8 (subgroup of HD-ZIP III) gene resulted in precocious proliferation of xylem tissue while absence of vascular phenotype in their knockout cannot be explained by redundancy among the HD-ZIP III members [8], [15]. Overexpression of sense and antisense ATHB15 (subgroup of HD-ZIP III) transcripts resulted in moderately dwarfed and severely dwarfed transgenic plants which may due to the suppressed vascular system [9]. ATHB15 negatively regulate cell differentiation and is critical for vascular development [9]. Xylem formation are no longer detected in root when all the five HD-ZIP III transcription factors are knocked out as this group of transcription factor drives the de novo xylem formation [45], [46]. In agreement with Carlsbecker et al. [45] and Cano-Delgado et al. [46], our study suggest that amg-miR166 may play a potential role in xylem formation as the expression of this miRNA family was strongly downregulated in xylem tissue, accompanied by the upregulation of HD-ZIP transcription factors (Figure 1 and 4). In the March issue of Plant & Cell Physiology, Zhong and Ye [15] reported that overexpression of miR165 leads to the downregulation of all the five HD-ZIP ΙΙΙ transcript genes. Although HD-ZIP ΙΙΙ transcripts are regulated by miR165 and miR166 [15], our deep sequencing study reported the absence of miR165 member in low lignin Am54 and high lignin Am48 [12].

### Spatial and temporal expression of highly conserved miRNA families

Our results revealed the involvement of a cascade of miRNAs in the regulatory network in secondary wall formation in *A. mangium*. Experimental analysis suggests that temporal expression of amg-miR168, amg-miR159, amg-miR172, amg-miR156 and amg-miR394 in compression wood and tension wood indirectly assisting the role of amg-miR166 (Figure 1). These six amg-miRNA members demonstrated obvious differences in the intensity between high lignin compression wood and low lignin tension wood. However, only amg-miR166 demonstrated obvious differences in the intensity between phloem and xylem tissues, suggesting its roles in xylem development (Figure 1). As flowering plays a pivotal role in xylem expansion [47], differential expression of the miRNA members in this study annotated with flowering properties (amg-miR159, amg-miR156 and ang-miR172) suggest its complexity in plant growth and development. Secondary wall formation is very complex as it involves various groups of regulatory network with some families playing dual roles by regulating the synthesis of cellulose and lignin simultaneously. These findings suggest that the five different amg-miRNA play dual roles in secondary wall formation.

Lu et al. [11] reported that a large number of highly conserved miRNA families exhibit contrasting species-tissue-specific expression patterns in *Arabidopsis* and *Populus* despite of sequence conservation between these two species. In this study, amg-miR156, amg-miR159, amg-miR172, amg-miR394, amg-miR168 and amg-miR166 have the most conspicuous expression in tension wood compared to compression wood (Figure 1). These miRNA families were also abundantly expressed in tension wood compared to compression wood [11]. Although miR156 family showed tissue specific expression level in *Populus*, *Arabidopsis* and in this study, however, this miRNA family was expressed to a similar level in all tissues in apple [13]. Expression of ptr-miR159 was significantly higher in compression wood than in tension wood with no preferential differences in expression between phloem and xylem tissue [11]. In apple stem, this miRNA family was weakly expressed in xylem tissue compared to phloem tissue [13].

We hypothesized that miRNAs play an independent and additive role during secondary wall formation with the expression of some miRNAs indirectly affecting the expression level of other downstream miRNAs. It is evident from the expression pattern of various miRNA members chosen for validation in this study (Figure 1). Spatial and temporal expression of these miRNA members in leaf, phloem, compression wood and tension wood tissue is likely to fine-tune the expression of target transcripts beyond a certain level (Figure 1, 3 and 4). For instance, amg-miR166 was highly expressed in leaf and phloem tissue and only mild expression was detected in xylem tissue. These differential expression patterns of amg-miR166 in different tissues suggest downregulation of this amg-miR166 family being important for HD-ZIP ΙΙΙ transcription factors playing a role in xylem development and indirectly regulate the expression of various pathway genes involved in lignin biosynthesis. Studies by Ohashi-Ito et al. [17] revealed that overexpression with a mutation in the START domain of Ze-HB12 (HD-ZIP III, zinnia homologue of *Arabidopsis* REVOLUTA) triggered the upregulation of four genes encoding enzymes involved in lignin monomer synthesis. Du et al. [16] reported overexpression of a miRNA-resistant poplar HD-ZIP III (POPCORONA, poplar homologue of ATHB15) resulting in the up-regulation of genes involved in cellulose biosynthesis while lignification and metabolite related genes (putative laccase, cinnamoyl CoA reductase, chalcone synthase, putative pectin methyltransferases and pectinesterase) are downregulated. Overexpression of ATHB8 in *Arabidopsis* promotes vascular cell differentiation and accompanied with an increased production of lignified tissues [8]. Our study suggest the involvement of other highly conserved amg-miRNAs which indirectly regulate the expression of various pathways genes involved in secondary wall biosynthesis from their expression patterns in compression wood and tension wood. Expression of lignin pathway genes are reported to be regulated by various transcription factors [16], [17], [48], [49], [50], [51], [52].

### MicroRNA plays an important regulatory role in secondary wall formation

Lignin formation involves complex interaction between various groups of transcription factors with the monolignol pathway genes. Various groups of transcription factors as MYB, NAC, LIM and HD-ZIP ΙΙΙ were reported to independently regulate the expression of various pathway genes in lignin biosynthesis [6], [8], [9], [52], [53]. Studies in models species have validated the roles of highly conserved miRNAs with transcription factors target in regulating the monolignol biosynthetic pathway genes which suggest that the regulatory mechanism in vascular development and lignin biosynthesis might be conserved in diverse plants species [54]. This effect was seen very clearly in the expression profile of various amg-miRNA families in various tissues (Figure 1), low lignin Am54 and high lignin Am48 (Table S1).

Better understanding of the biological functions of miRNAs requires knockdown of their mRNA target. Various knockdown studies have revealed that both miRNA and their target genes exhibited negative correlation [55], [56], [57] and this implies that expression of miRNA is indeed an indicator of their target abundance. In line with this, our analysis proves that a single miRNA can regulate the stability of several different transcripts from a particular family (Figure 3). *In vitro* assay of *Arabidopsis* miRNA gene target via bioinformatics approach indicated that distantly related transcripts can have the same miRNA target site demonstrating expression of miRNA being best to reflect the regulatory events in secondary wall formation (Figure S2). In *Arabidopsis*, all the class ΙΙΙ HD-ZIP members display overlapping and distinct roles in vascular development [58], [59], [60], [61] although all these different members were regulated by miR166. Interestingly, 5′RACE mapping of our amg-miR166 target cleavage site revealed this miRNA family has nine different transcripts.

Although MYB are the most investigated transcription factor in the regulation of lignin biosynthetic pathway genes, antagonistic and additive roles of the various members belonging to this family have added additional complexity to the transcriptional regulation of monolignol biosynthetic pathway. For instance, AtMYB58, AtMYB63 and AtMYB85 are identified as the transcriptional activators of lignin biosynthesis in vessels and fibers [2], [62] while AtMYB103, in cellulose biosynthesis [2]. These antagonistic roles were complicated with the discovery of other MYB members like AtMYB52, AtMYB54, and AtMYB69 that regulate the entire cellulose, lignin and xylan biosynthesis [2]. In case of miR159 family, we showed that six different MYB members are regulated by amg-miR159 (Figure S2).

Our analysis on the expression of these various amg-miRNA families and indirect *in vitro* assay via bioinformatics approach suggests miRNA being involved in fine tuning the expression of its transcripts via RNAi and epigenetic silencing. To gain more insight into this mechanism, we have shown in our RT-qPCR the miRNA members and their corresponding HD-ZIP ΙΙΙ gene targets were inversely expressed in various tissues (Figure 1 and 4). Downregulation of these amg-miRNA members in compression wood is critical for transcriptional regulation of various pathway genes involved in secondary wall biosynthesis. This was evident from expression of *C4H*, *CCoAOMT* and *CAD* genes in compression wood, a region enriched with lignin content in this study.

### Complexity in the regulatory network in monolignol biosynthetic pathway

Wood (secondary xylem) formation involves various stages of division zone, expansion zone, maturation zone and programme cell death zone [63]. Various transcription factors are implicated in the regulation of cell division, differentiation, expansion and patterning of vascular tissue during secondary wall formation [64], [65], [66], [67], [68], [69]. This effect was seen very clearly in the expression profile of various amg-miRNA families in various tissues (Figure 1), low lignin Am54 and high lignin Am48 [12]. Moreover, this observation raise important questions on the complexity involved in lignin regulation as various groups of short small RNAs with very different expression level were reported between low lignin Am54 and high lignin Am48 (data not shown).

For comparison, Oh et al. [70] showed that transcription factors were highly expressed in xylem tissue which explains the events where secondary xylem formation occurs. Xylem-abundant MYB proteins are involved in the transcriptional regulation of secondary xylem formation from their upregulation in the xylem tissue compared to the bark tissue [70], [71]. In contrast to class Ι KNOX genes in negatively regulating the differentiation of cambial cells, transcription factors like NAC domain promote differentiation of vessel elements [6], [72], [73]. Hence, it is obvious that regulatory network in monolignol biosynthetic pathway involves a complex interaction of various groups of transcription factors.

Since the lignin biosynthetic pathway genes are more or less highly conserved across all plant species, it is likely a common mechanism regulating vascular tissue development in all plants [10]. This is evident from studies reported on the roles of miR166 in vascular differentiation and development via HD-ZIP III genes; a possible general rule in all vascular plants [9]. In addition, miR166 and its target genes HD-ZIP III are ancient and the most well studied small RNA in plants [8], [58], [59], [60], [74], [75]. In agreement with many other studies, our findings suggest amg-miR166 play a potential roles in xylem development. Moreover, members of this amg-miR166 family regulate different HD-ZIP III transcripts during xylem development. In addition to amg-miR166, our study suggests secondary wall biosynthesis may involve other highly conserved miRNA families like amg-miR394, amg-miR159, amg-miR156, amg-miR172 and amg-miR168.

### Inconsistency in findings on alteration of the pathway genes

Although much attention has been devoted into isolation and characterization of all the genes involved in monolignol biosynthetic pathway, many fundamental questions about their regulation remain unanswered. Different laboratories reported conflicting results on alteration of the pathway genes even within a similar species. These complexity raise questions on our understanding in the regulatory network involved in monolignol biosynthetic pathway as various groups of transcription factors have been shown to regulate the pathway genes.

System biology has documented that altering the pathway genes often modifies its interaction with the environment [76], [77]. These were true from the contrasting reports on the effects of downregulation of 4CL via antisense RNA on the growth performance. Hu et al. [25] reported greenhouse evaluation of suppressed 4CL activity exhibiting up to a 45% reduction in lignin and these were compensated with substantially enhanced leaf, root and stem growth in transgenic poplar. Subsequent studies found no growth enhancement using transgenes suppressing the expression of 4CL in Chinese white poplar (*P. tomentosa*) [78] and greenhouse grown aspen [79], [80], [81]. However, 4CL downregulation in poplar grown under field condition resulted in considerable variation in productivity [82]. In agreement with Pilate et al. [83] and Leple et al. [84], Voelker et al. [82] reported that high level of lignin reduction observed in approximately one-third of the transgenic events led to reduced growth and serious physiological abnormalities.

Since different research groups reported inconsistent findings on the effects of downregulation of pathway genes even within same model species, our study suggest better understanding of the roles of miRNA is critical as miRNA were reported to regulate various transcripts with overlapping roles, which in return regulate the monolignol biosynthetic pathway genes. Thus, we believe that the findings presented in this study will add more blocks to the network and expand the knowledge on the underlying regulatory details as the major pathways in lignin biosynthesis are more or less conserved across various species.

## Methods

### Plant materials

Plant materials for gene expression analysis and lignin content determination were used from three 4-year-old trees of *A. mangium* from Plot A, Plant Biotechnology Centre, Universiti Kebangsaan Malaysia, Bangi, Malaysia. Three seed sources (NSW19, NSW20 and ERC22) were used in this investigation. Four different types of tissue- leaf, phloem, compression wood and tension wood- were used in this investigation. Compression wood was taken from the lower portion of the bend and tension wood was collected from the upper portion of the bend. Phloem tissues were taken from the outer layer of the inner bark tissues of the main stem.

### Quantitative real time PCR of selected miRNAs

Quantitative real time PCR was performed using iCycler iQ™ Real Time PCR Detection System (Biorad, USA). From the expression of 12 highly conserved amg-miRNAs between low lignin Am54 and high lignin Am48 [12], we have identified six highly conserved amg-miRNAs (Table S1) play an important regulatory roles in secondary wall formation. Expression analysis using semi-qPCR and rt-qPCR indicated the remaining six amg-miRNAs are lowly expressed and no obvious differences were observed between high lignin compression wood and low lignin tension wood (Figure S3 for semi-qPCR, method unpublished). From here, one miRNA from each family with strong differences in expression between low lignin Am54 and high lignin Am48 (Table S1) were selected for further validation in various tissues using iQ™ SYBR Green Supermix (Biorad, USA) among three different individuals. Total RNA containing small RNA was isolated from four different tissues using miRVana miRNA Isolation kit (Ambion, Austin, TX, USA) and treated with RNase-free DNase (Qiagen, Germany). Isolated Total RNA was analyzed for their integrity using RNA 6000 Nano kit (Agilent Bioanalyzer, USA) and Nanodrop ND-100 Spectrophotometer. Only Total RNA with RIN value above 7.5 was selected for the first strand cDNA synthesis. Total RNA containing small RNA was reverse transcribed using miScript Reverse Transcription kit (Qiagen, Germany). 5.8 S rRNA was used to normalize the cDNA concentration in various tissues. In each reaction, 15 µl consisting of 6 µl of 2× SYBR Green Supermix (Biorad, USA), 1 µl of cDNA template equivalent to 100 pg total RNA, and 2 µl of 10× miScript Universal Primer (Qiagen, Germany) and 5 pmol of forward primers. PCR amplification protocol was as follows: 95°C for PCR initiation activation step, 45 cycles consisting of 15 s at 94°C, 30 s at 57°C and 30 s at 70°C and final extension of 4 min at 70°C.

### Lignin content determination

The lignin content of compression and tension wood tissues from three different *A. mangium* trees were determined using pyrolysis gas chromatography mass spectrometry (GC-MS) method. Each analysis was done in triplicates. Dried wood samples were ground to fine powder using a electric blender. Extractive content in wood meal was removed by refluxing them with 2 volumes of toluene and 1 volume of ethanol for 8 hours at 6 volts. Later on, the wood meal was refluxed with ethanol for 4 h to remove any residue of toluene. Pyrolysis GCMS was performed using GCMS G1374A (Agilent, USA). Compounds were identified by their mass spectra and retention time through comparison with those reported in literature [85], [86]. Total lignin was calculated by adding all the S, G and H units.

### miRNA: mRNA target prediction

miRNA: mRNA duplexes of all the isoforms belonging to amg-miR166 were characterized using psRNA Target Analysis Server [87]. In these analyses, scoring systems for mismatches in the miRNA: mRNA were as follow; 0 score assigned to a complementary pair, G:U wobble assigned 0.5 score, non-G:U wobble mismatches assigned 1 score and finally 2 scores assigned to each bulged nucleotide [40], [87], [88], [89]. miRNA: mRNA duplex with scoring of 3.5 or less were considered high confidence [40], [87], [88], [89]. Nine different HD-ZIP III mRNAs isolated using 5′RACE were characterized for their binding energy with the five different members of amg-miR166. In addition, HD-ZIP III sequences of ten different species were downloaded from National Center for Biotechnology Information (NCBI) and used to predict their binding energies with amg-miR166. psRNA Target Analysis and binding energy were conducted with default settings. Subsequent miRNA:mRNA analysis was done using various members with overlapping roles from *Arabidopsis* with each different highly conserved amg-miRNA (amg-miR159, amg-miR156, amg-miR168, amg-miR172 and amg-miR394).

### 3′ and 5′ Rapid Amplification of cDNA Ends (RACE) for mapping of amg-miR166 cleavage site

Aliquots of total RNA from compression wood and tension wood from three 4-year-old individuals of *A. mangium*, pooled prior to first strand cDNA synthesis. First strand cDNA was constructed using SMART™ RACE cDNA Amplification Kit (CLONTECH Laboratories, Inc. USA) following protocol instruction. 3′ RACE was first employed to obtain the gene target of amg-miR166. In the 3′RACE PCR amplification, primer sequence flanking the amg-miR166 target site was designed (Table S2). About 20 clones were randomly selected and were sequenced. To amplify the 5′ ends of the target miRNA, two amplifications were performed using the 3′RACE sequence (Table S2). The outer primer sequence of 3′RACE was used in the first 5′RACE PCR amplification. The 5′RACE amplified products were purified and re-amplified using the inner 3′RACE sequence in the following 5′RACE PCR amplification (Table S2). In addition, amg-miR166 sequence ID 256(21) was used to obtain the novel HD-ZIP III (HD-ZIP (5–9) in 5′ mapping of amg-miR166 cleavage site. The amplified products were gel purified, cloned into pGEM-T vector, sequenced and analyzed. To obtain the full lengths of the highly conserved HD-ZIP ΙΙΙ mRNA (HD-ZIP (1–4), forward and reverse primers from each of the different HD-ZIP ΙΙΙ mRNA were designed from the partial RACE HD-ZIP ΙΙΙ sequence. First strand cDNA was synthesized using SMART™ RACE cDNA Amplification Kit (CLONTECH Laboratories, Inc. USA) and was used to obtain the full length of the different HD-ZIP ΙΙΙ sequences in the three different *A. mangium trees*. 5′ RACE mapping of the cleavage site were then employed in each of trees using leaf, phloem, pooled compression wood and tension wood tissues.

### RT-qPCR of HD-ZIP III transcription factors and key lignin genes

Quantitative real time – PCR was performed using iQ™ SYBR Green Supermix (Biorad, USA) in iCycler iQ™ Real Time PCR Detection System (Biorad, USA) in a final volume of 15 µl. PCR primers (Table S3) were designed from the RACE sequence. The PCR protocol employed was the same as in quantitative real time PCR analysis of the selected miRNAs. Actin gene was included as an internal control for normalization in cDNA amounts used. The reactions were performed in triplicate for each run with three biological replicates. Melt curve analysis was conducted to determine the amplification specificity and the amplified product was further analyzed in 2% agarose. Relative normalized expression of the four different HD-ZIP ΙΙΙ and *C4H*, *CCoOAMT* and *CAD* genes in leaf, phloem, compression wood and tension wood were compared.

### Precursor amg-miR166 isoforms characterization

We designed primers flanking the conserved region of mature miRNA and the opposite arm from precursor structure sequence available in miR Base (http://microrna.sanger.ac.uk/). *Arabidopsis* and *Poplar* miRNA precursor databases from miR Base were used in the primer design. In addition, we used all five different amg-miR166 sequences as forward and reverse sequences to obtain a variety of precursors structure as miRNA and miRNA* often have quite perfect matches. PCR was conducted using DNA from *A. mangium* NSW19 with amplification protocol as follows: 95°C for PCR initiation activation step, 35 cycles consisting of 45 s at 94°C, 30 s at 50°C and 30 s at 70°C and final extension of 4 min at 70°C. Primers used are listed in Table S4. For each PCR, 20 µl reaction volumes consist of 1 U/µl i-Taq DNA polymerase (intron), 2 µl 10× PCR buffer (intron), 2 µl dNTP mixture (intron), 1 µl of DNA equivalent to 100 ng and 2 µl mixture of 10 µM forward and reverse primer respectively. PCR products were gel purified, cloned into pGEM-T vector and sequenced. The sequences obtained were analyzed for their ability to fold into hairpin structures using Mfold 3.1 [90], [91] with default settings.

### Conclusion

Our study critically demonstrated the roles of highly conserved miRNAs in secondary wall formation. From the expression pattern of six different highly conserved amg-miRNA families in various tissues, only amg-miR166 was strongly downregulated in xylem tissue compared to phloem tissue across six different genotypes. This phenomenon suggests that amg-miR166 might play a potential role in xylem development and indirectly regulate the genes involved in lignin biosynthesis in *Acacia mangium*. Moreover, this miRNA family was found in all vascular plants. Since different research groups have reported inconsistent findings on the effects of downregulation of pathway genes even within same model species, this study provides a better understanding of the overlapping roles of miRNA in regulating the pathway genes involved in secondary wall formation.

## Supporting Information

Figure S1 **Nucleotide sequence of the nine mRNA target of amg-miR166 obtained via 3′ and 5′ RACE.**
(DOC)

Figure S2 **Binding energy characterization between amg-miRNA members with various mRNA isoforms from** ***Arabidopsis*** **.**
(DOC)

Figure S3 **Semi-quantitative reverse transcription PCR analysis of 12 highly conserved miRNA families in Leaf (L), Phloem (P), Xylem (X), Compression Wood (CW) and Tension Wood (TW).**
(DOC)

Table S1 The 6 highly conserved plant miRNA families with strong differences in the expression level in each of the isoforms between low lignin Am54 and high lignin Am48.
(DOC)

Table S2 Primers used for 5′ and 3′ mapping of the amg-miR166 cleavage sites.
(DOC)

Table S3 List of the forward and reverse primers used in RT-qPCR of various HD-ZIP III and three key lignin genes in leaf, phloem, compression wood and tension wood.
(DOC)

Table S4 List of the forward and reverse primers used in amg-miR166 precursors structure characterization.
(DOC)

## References

[1] Plomion C, Leprovost G, Stokes A (2001) Wood formation in trees. Plant Physiol 127: 1513–1523. 11743096 PMC1540185

[2] Zhong R, Lee C, Zhou J, McCarthy RL, Ye ZH (2008) A Battery of Transcription Factors Involved in the Regulation of Secondary Cell Wall Biosynthesis in *Arabidopsis* . Plant Cell 20: 2763–2782. 18952777 10.1105/tpc.108.061325 PMC2590737

[3] Boerjan W, Ralph J, Baucher M (2003) Lignin biosynthesis. Annu Rev Plant Biol 54: 519–546. 14503002 10.1146/annurev.arplant.54.031902.134938

[4] Mellerowicz EJ, Sundberg B (2008) Wood cell walls: biosynthesis, developmental dynamics and their implications for wood properties. Curr Opin Plant Biol 11: 293–300. 18434240 10.1016/j.pbi.2008.03.003

[5] Zhao Q, Dixon RA (2011) Transcriptional networks for lignin biosynthesis: more complex than we thought? Trends in Plant Science 16: 227–233. 21227733 10.1016/j.tplants.2010.12.005

[6] Zhong R, Ye ZH (2009) Transriptional regulation of lignin biosynthesis. Plant Signal Behav 4: 1028–1034. 19838072 10.4161/psb.4.11.9875 PMC2819510

[7] Ambavaram MMR, Krishnan A, Trijatmiko KR, Pereira A (2011) Coordinated Activation of Cellulose and Repression of Lignin Biosynthesis Pathways in Rice. Plant Physiol 155: 916–931. 21205614 10.1104/pp.110.168641 PMC3032476

[8] Baima S, Possenti M, Matteucci A, Wisman E, Altamura MM, et al (2001) The *Arabidopsis* ATHB-8 HD-ZiP Protein Acts as a Differentiation-Promoting Transcription factor of the Vascular Meristems. Plant Physiol 126: 643–655. 11402194 10.1104/pp.126.2.643 PMC111156

[9] Kim J, Jung JH, Reyes JL, Kim YS, Kim SY, et al (2005) MicroRNA-directed cleavage of ATHB15 mRNA regulates vascular development in *Arabidopsis* inflorescence stems. Plant J 42: 84–94. 15773855 10.1111/j.1365-313X.2005.02354.x PMC1382282

[10] Victor M, Myburg AA, Huismans H (2006) MicroRNAs in differentiating tissues of *Populus* and *Eucalyptus* trees. University of Pretoria dissertation

[11] Lu S, Sun YH, Shi R, Clark C, Li L, et al (2005) Novel and mechanical stress-responsive microRNAs in *Populus trichocarpa* that are absent from *Arabidopsis* . Plant Cell 17: 2186–2203. 15994906 10.1105/tpc.105.033456 PMC1182482

[12] Ong SS, Wickneswari R (2011) Expression Profile of small RNAs in *Acacia mangium* Secondary Xylem Tissues with Contrasting Lignin Content – potential regulatory sequences in monolignol biosynthetic pathway. BMC Genomics 12: S13. 10.1186/1471-2164-12-S3-S13 PMC3333172 22369296

[13] Varkonyi-Gasic E, Gould N, Sandanayaka M, Sutherland P, MacDiarmid RM (2010) Characterisation of microRNAs from apple (*Malus domestica ‘Royal Gala’*) vascular tissue and phloem sap. BMC Plant Biol 10: 159. 20682080 10.1186/1471-2229-10-159 PMC3095296

[14] Ko JH, Prassinos C, Han KH (2006) Developmental and seasonal expression of PtaHB1, a *Populus* gene encoding a class III HD-Zip protein, is closely associated with secondary growth and inversely correlated with the level of microRNA (miR166). New Phytol 169: 469–478. 16411950 10.1111/j.1469-8137.2005.01623.x

[15] Zhong R, Ye ZH (2007) Regulation of HD-ZIP III genes by MicroRNA 165. Plant Signal Behav 2: 351–353. 19704656 10.4161/psb.2.5.4119 PMC2634209

[16] Du J, Miura E, Robischon M, Martinez C, Groover A (2011) The Populus Class III HD ZIP Transcription Factor POPCORONA Affects Cell Differentiation during Secondary Growth of Woody Stems. PLoS ONE 6 (2) e17458 doi:10.1371/journal.pone.0017458 21386988 10.1371/journal.pone.0017458 PMC3046250

[17] Ohashi-Ito K, Kubo M, Demura T, Fukuda H (2005) Class III Homeodomain Leucine-Zipper Proteins Regulate Xylem Cell Differentiation. Plant Cell Physiol 46: 1646–1656. 16081527 10.1093/pcp/pci180

[18] Boualem A, Laporte P, Jovanovic M, Laffont C, Plet J, et al (2008) MicroRNA166 controls root and nodule development in *Medicago truncatula* . Plant J 54: 876–887. 18298674 10.1111/j.1365-313X.2008.03448.x

[19] Song C, Fang J, Wang C, Guo L, Nicholas KK, et al (2010) MiR-RACE, a New Efficient Approach to Determine the Precise Sequences of Computationally Identified Trifoliate Orange (*Poncirus trifoliata*) MicroRNAs. PLoS ONE 5 (6) e10861 doi:10.1371/journal.pone.0010861 20539756 10.1371/journal.pone.0010861 PMC2881865

[20] Bonnet E, Wuyts J, Rouze P, Van de Peer Y (2004) Detection of 91 potential conserved plant microRNAs in *Arabidopsis thaliana* and *Oryza sativa* identifies important target genes. Proc Natl Acad Sci USA 101: 11511–11516. 15272084 10.1073/pnas.0404025101 PMC509231

[21] Zhang Y (2005) miRU: an automated plant miRNA target prediction server. Nucleic Acids Res 33: 701–704. 10.1093/nar/gki383 PMC1160144 15980567

[22] Yong SYC, Choong CY, Cheong PL, Pang SL, Nor Amalina R, et al (2011) Analysis of ESTs generated from inner bark tissue of an *Acacia auriculiformis* x *Acacia mangium* hybrid. Tree Genetics & Genomes 7: 143–152.

[23] Pang SL (2008) Characterization and downregulation of lignin genes from *Acacia auriculiformis* x *Acacia mangium* hybrid. Universiti Kebangsaan Malaysia. PhD dissertation

[24] Andersson-Gunnerås S, Mellerowicz EJ, Love J, Segerman B, Ohmiya Y, et al (2006) Biosynthesis of cellulose- enriched tension wood in Populus: global analysis of transcripts and metabolites identifies biochemical and developmental regulators in secondary wall biosynthesis. Plant J 45: 144–165. 16367961 10.1111/j.1365-313X.2005.02584.x

[25] Hu WJ, Lung J, Harding SA, Popko JL, Ralph J, et al (1999) Repression of lignin biosynthesis promotes cellulose accumulation and growth in transgenic trees. Nat Biotechnol 17: 808–812. 10429249 10.1038/11758

[26] Jin H, Kwon M (2009) Mechanical bending induced tension wood formation with reduced lignin biosynthesis in Liriodendron tulipifera. J Wood Sci 55: 401–408.

[27] Lohrasbei H, Mabbee E, Royd DN (1999) Chemistry and pulping feasibility of compression wood in black spruce. J Wood Chem Tech 19: 13–25.

[28] Moon D, Shin SJ, Choi JW, Park JS, Kim W, et al (2011) Chemical modification of secondary xylem under tensile stress in the stem of Liriodendron tulipifera. Forest Sci Technol 7: 53–59.

[29] Novaes E, Kirst M, Chiang V, Winter-Sederoff H, Sederoff R (2010) Lignin and Biomass: A Negative Correlation for Wood Formation and Lignin Content in Trees. Plant Physiol 154: 555–561. 20921184 10.1104/pp.110.161281 PMC2949025

[30] Pilate G, Chabbert B, Cathala B, Yoshinaga A, Leplé JC, et al (2004) Lignification and tension wood. C R Biol 327: 889–901. 15587080 10.1016/j.crvi.2004.07.006

[31] Timell TE (1986) Compression Wood in Gymnosperms. Berlin: Springer-Verlag. p. 1338.

[32] Timell TE (1982) Recent progress in the chemistry and topochemistry of compression wood. Wood Sci Technol 16: 83–122.

[33] Yeh TF, Goldfarb B, Chang HM, Peszlen I, Braun JL, et al (2005) Comparison of morphological and chemical properties between juvenile wood and compression wood of loblolly pine. Holzforschung 59: 669–674.

[34] Wang HZ, Dixon RA (2012) On-Off Switches for Secondary Cell Wall Biosynthesis. Molecular Plant 5: 297–303. 22138968 10.1093/mp/ssr098

[35] Chen X (2005) MicroRNA biogenesis and function in plants. FEBS Lett 31: 5923–5931. 10.1016/j.febslet.2005.07.071 PMC5127707 16144699

[36] Bernstein E, Caudy AA, Hammond SM, Hannon GJ (2001) Role for a bidentate ribonuclease in the initiation step of RNA interference. Nature 409: 363–366. 11201747 10.1038/35053110

[37] Kim VN (2005) MicroRNA biogenesis and coordinated cropping and dicing. Nat Rev Mol Cell Biol 6: 376–85. 15852042 10.1038/nrm1644

[38] Rana TM (2007) Illuminating the silence: understanding the structure and function of small RNAs. Nat Rev Mol Cell Biol 8: 23–36. 17183358 10.1038/nrm2085

[39] Krol J, Sobczak K, Wilczynska U, Drath M, Jasinska A, et al (2004) Structural Features of MicroRNA (miRNA) Precursors and Their Relevance to miRNA Biogenesis and Small Interfering RNA/Short Hairpin RNA Design. J Biol Chem 279: 42230–42239. 15292246 10.1074/jbc.M404931200

[40] Jones-Rhoades MW, Bartel DP (2004) Computational identification of plant microRNAs and their targets, including a stress-induced miRNA. Mol. Cell 14: 787–799. 10.1016/j.molcel.2004.05.027 15200956

[41] Bonnet E, Wuyts J, Rouze P, Van de Peer Y (2004) Evidence that microRNA precursors, unlike other non-coding RNAs, have lower folding free energies than random sequences. Bioinformatics 20: 2911–2917. 15217813 10.1093/bioinformatics/bth374

[42] Legrand S, Valot N, Nicole F, Moja S, Baudino S, et al (2010) One-step identification of conserved miRNAs, their targets, potential transcription factors and effector genes of complete secondary metabolism pathways after 454 pyrosequencing of calyx cDNAs from the Labiate Salvia sclarea L. Gene 450: 55–62. 19840835 10.1016/j.gene.2009.10.004

[43] Turner S, Gallois P, Brown D (2007) Tracheary Element Differentiation. Annu Rev Plant Biol 58: 407–433. 17472568 10.1146/annurev.arplant.57.032905.105236

[44] Bao N, Lye KW, Barton MK (2004) MicroRNA binding sites in *Arabidopsis* class III HD-ZIP mRNA are required for methylation of the template chromosome. Dev Cell 7: 653–662. 15525527 10.1016/j.devcel.2004.10.003

[45] Carlsbecker A, Lee JY, Roberts CJ, Dettmer J, Lehesranta S, et al (2010) Cell signalling by microRNA165/6 directs gene dose-dependent root cell fate. Nature 465: 316–21. 20410882 10.1038/nature08977 PMC2967782

[46] Cano-Delgado AC, Lee JY, Demura T (2010) Regulatory Mechanisms for Specification and Patterning of Plant Vascular Tissues. Annu Rev Cell Dev Biol 26: 605–637. 20590454 10.1146/annurev-cellbio-100109-104107

[47] Sibout R, Plantegenet S, Hardtke CS (2008) Flowering as a condition for xylem expansion in *Arabidopsis* hypocotyl and root. Curr Biol 18: 458–463. 18356049 10.1016/j.cub.2008.02.070

[48] Patzlaff A, McInnis S, Courtenay A, Surman C, Newman LJ, et al (2003) Characterization of a pine MYB that regulates lignification. Plant J 36: 743–754. 14675440 10.1046/j.1365-313x.2003.01916.x

[49] Borevitz JO, Xia Y, Blount J, Dixon RA, Lamb C (2000) Activation tagging identifies a conserved MYB regulator of phenylpropanoid biosynthesis. Plant Cell 12: 2383–2393. 11148285 10.1105/tpc.12.12.2383 PMC102225

[50] Goicoechea M, Lacombe E, Legay S, Mihaljevic S, Rech P, et al (2005) EgMYB2, a new transcriptional activator from Eucalyptus xylem, regulates secondary cell wall formation and lignin biosynthesis. Plant J 43: 553–567. 16098109 10.1111/j.1365-313X.2005.02480.x

[51] McCarthy RL, Zhong R, Ye ZH (2009) MYB83 Is a direct target of SND1 and acts redundantly with MYB46 in the regulation of secondary cell wall biosynthesis in *Arabidopsis* . Plant and Cell Physiol 50: 1950–1964. 19808805 10.1093/pcp/pcp139

[52] Bomal C, Bedon F, Caron S, Mansfield SD, Levasseur C, et al (2008) Involvement of *Pinus taeda MYB1* and *MYB8* in phenylpropanoid metabolism and secondary cell wall biogenesis: a comparative in planta analysis. J of Exp Bot 59: 3925–3939. 18805909 10.1093/jxb/ern234 PMC2576632

[53] Han KH, Ko JH, Yang SH (2007) Optimizing lignocellulosic feedstock for improved biofuel productivity and processing. Biofuels Bioprod Bioref 1: 135–146.

[54] Zhang J, Elo A, Helariutta Y (2010) *Arabidopsis* as a model for wood formation. Curr Opin in Biotech 22: 1–7. 10.1016/j.copbio.2010.11.008 21144727

[55] Nicolas FE, Pais H, Schwach F, Lindow M, Kauppinen S, et al (2008) Experimental identification of microRNA-140 targets by silencing and overexpressing miR-140. RNA 14: 2513–2520. 18945805 10.1261/rna.1221108 PMC2590970

[56] Aukerman MJ, Sakai H (2003) Regulation of Flowering Time and Floral Organ Identity by a MicroRNA and Its APETALA2 -Like Target Genes. Plant Cell 15: 2730–2741. 14555699 10.1105/tpc.016238 PMC280575

[57] Chen C, Ridzon DA, Broomer AJ, Zhou Z, Lee DH, et al (2005) Real time quantification of microRNAs by stem loop RT-PCR. Nucleic Acids Res 33: 179. 10.1093/nar/gni178 PMC1292995 16314309

[58] Prigge MJ, Otsuga D, Alonso JM, Ecker JR, Drews GN, et al (2005) Class III Homeodomain-Leucine Zipper Gene Family Members Have Overlapping, Antagonistic, and Distinct Roles in *Arabidopsis* Development. Plant Cell 17: 61–76. 15598805 10.1105/tpc.104.026161 PMC544490

[59] Green KA, Prigge MJ, Katzman RB, Clark SE (2005) CORONA, a Member of the Class III Homeodomain Leucine Zipper Gene Family in *Arabidopsis*, Regulates Stem Cell Specification and Organogenesis. Plant Cell 17: 691–704. 15705957 10.1105/tpc.104.026179 PMC1069692

[60] McConnell JR, Emery JF, Eshed Y, Bao N, Bowman J, et al (2001) Role of PHABULOSA and PHAVOLUTA in determining radial patterning in shoots. Nature 411: 709–712. 11395776 10.1038/35079635

[61] Emery JF, Floyd SK, Alvarez J, Eshed Y, Hawker NP, et al (2003) Radial patterning of *Arabidopsis* shoots by class III HD-ZIP and KANADI genes. Curr Biol 13: 1768–1774. 14561401 10.1016/j.cub.2003.09.035

[62] Zhou J, Lee C, Zhong R, Ye ZH (2009) MYB58 and MYB63 are transcriptional activators of the lignin biosynthetic pathway during secondary cell wall formation in *Arabidopsis* . Plant Cell 21: 248–266. 19122102 10.1105/tpc.108.063321 PMC2648072

[63] Chaffey N (1999) Cambium: old challenges–new opportunities. Trees 13: 138–151.

[64] Hagen G, Guilfoyle T (2002) Auxin-responsive gene expression: genes, promoters and regulatory factors. Plant Mol Biol 49: 373–385. 12036261

[65] Reed JW (2001) Roles and activities of Aux/IAA proteins in *Arabidopsis* . Trends Plant Sci 6: 420–425. 11544131 10.1016/s1360-1385(01)02042-8

[66] Sundberg B, Ugglar C, Tuominen H (2000) Cambial growth and auxin gradient. In: Savidge RA, Barnett JR, Napier R, editors. Cell and molecular biology of wood formation. Oxford, UK: Bios Scientific Publishers Ltd.

[67] Groover AT (2005) What genes make a tree? Trends Plant Sci 10: 210–214. 15882652 10.1016/j.tplants.2005.03.001

[68] Spicer R, Groover A (2010) The evolution of development of the vascular cambium and secondary growth. New Phytol 186: 577–592. 20522166 10.1111/j.1469-8137.2010.03236.x

[69] Schrader J, Nilsson J, Mellerowicz E, Berglund A, Nilsson P, et al (2004) A high resolution transcript profile across the wood-forming meristem of poplar identifies potential regulators of cambial stem cell identity. Plant Cell 16: 2278–2292. 15316113 10.1105/tpc.104.024190 PMC520933

[70] Oh S, Park S, Han KH (2003) Transcriptional regulation of secondary growth in *Arabidopsis thaliana* . J of Exp Botany 54: 2709–2722. 14585825 10.1093/jxb/erg304

[71] Newman LJ, Campbell MM (2000) MYB proteins and xylem differentiation. In: Savidge RA, Barnett JR, Napier R, eds. Cell and molecular biology of wood formation. Oxford, UK: Bios Scientific Publishers Ltd. pp. 437–444.

[72] Du J, Mansfield SD, Groover AT (2009) The *Populus* homeobox gene ARBORKNOX2 regulates cell differentiation during secondary growth. Plant J 60: 1000–1014. 19737362 10.1111/j.1365-313X.2009.04017.x

[73] Groover A, Mansfield S, DiFazio S, Dupper G, Fontana J, et al (2006) The *Populus* homeobox gene ARBORKNOX1 reveals overlapping mechanisms regulating the shoot apical meristem and the vascular cambium. Plant Mol Biol 61: 917–932. 16927204 10.1007/s11103-006-0059-y

[74] Kidner CA, Martienssen RA (2004) Spatially restricted micro RNA directs leaf polarity through ARGONAUTE1. Nature 428: 81–84. 14999284 10.1038/nature02366

[75] McConnell JR, Barton MK (1998) Leaf polarity and meristem formation in *Arabidopsis* . Development 125: 2935–2942. 9655815 10.1242/dev.125.15.2935

[76] Vanholme R, Morreel K, Ralph J, Boerjan W (2008) Lignin engineering. Curr Opin Plant Biol 11: 278–285. 18434238 10.1016/j.pbi.2008.03.005

[77] Quentin M, Allasia V, Pegard A, Allais F, Ducrot PH, et al (2009) Imbalanced Lignin Biosynthesis Promotes the Sexual Reproduction of Homothallic Oomycete Pathogens. PLoS Pathog 5: e1000264 doi:10.1371/journal.ppat.1000264 19148278 10.1371/journal.ppat.1000264 PMC2613516

[78] Jia C, Zhao H, Wang H, Xing Z, Du K, et al (2004) Obtaining the transgenic poplars with low lignin content through down-regulation of 4CL. Chin Sci Bull 49: 905–90.

[79] Hancock JE, Bradley KL, Giardina CP, Pregitzer KS (2008) The influence of soil type and altered lignin biosynthesis on the growth and above and belowground biomass allocation of *Populus tremuloides* . Plant Soil 308: 239–253.

[80] Hancock JE, Loya WM, Giardina CP, Li L, Chiang VL, et al (2007) Plant growth, biomass partitioning and soil carbon formation in response to altered lignin biosynthesis in *Populus tremuloides* . New Phytol 173: 732–742. 17286822 10.1111/j.1469-8137.2006.01965.x

[81] Li L, Zhou Y, Cheng X, Sun J, Marita JM, et al (2003) Combinatorial modification of multiple lignin traits in trees through multigene cotransformation. Proc Natl Acad Sci USA 100: 4939–4944. 12668766 10.1073/pnas.0831166100 PMC153659

[82] Voelker SL, Lachenbruch B, Meinzer FC, Jourdes M, Ki C, et al (2010) Antisense down-regulation of 4CL expression alters lignification, tree growth and saccharification potential of field-grown poplar. Plant Physiol 110: 159–269. 10.1104/pp.110.159269 PMC2949011 20729393

[83] Pilate G, Guiney E, Holt K, Petit-Conil M, Lapierre C, et al (2002) Field and pulping performances of transgenic trees with altered lignification. Nat Biotechnol 20: 607–612. 12042866 10.1038/nbt0602-607

[84] Leplé JC, Dauwe R, Morreel K, Storme V, Lapierre C, et al (2007) Downregulation of cinnamoyl-coenzyme A reductase in poplar: Multiple-level phenotyping reveals effects on cell wall polymer metabolism and structure. Plant Cell 19: 3669–3691. 18024569 10.1105/tpc.107.054148 PMC2174873

[85] Ibarra D, del Rio JC, Gutierrez A, Rodriguez IM, Romero J, et al (2005) Chemical characterization of residual lignins from eucalypt paper pulps. J Anal Appl Pyrolysis 74: 116–122.

[86] Del Rio JC, Gutierrez A, Hernando M, Landin P, Romero J, et al (2005) Determining the influence of eucalypt lignin composition in paper pulp yield using Py-GC/MS. J Anal Appl Pyrolysis 74: 110–115.

[87] Dai X, Zhao PX (2011) psRNATarget: a plant small RNA target analysis server. Nucleic Acids Res 39: 155–159. 21622958 10.1093/nar/gkr319 PMC3125753

[88] Doench JG, Sharp PA (2004) Specificity of microRNA target selection in translational repression. Genes & Dev 18: 504–511. 15014042 10.1101/gad.1184404 PMC374233

[89] Valencia-Sanchez MA, Liu J, Hannon GJ, Parker R (2006) Control of translation and mRNA degradation by miRNAs and siRNAs. Genes & Dev 20: 515–524. 16510870 10.1101/gad.1399806

[90] Matthews DH, Sabina J, Zuker M, Turner DH (1999) Expanded sequence dependence of thermodynamic parameters improves prediction of RNA secondary structure. J Mol Bio 288: 911–940. 10329189 10.1006/jmbi.1999.2700

[91] Zucker M (2003) Mfold web server for nucleic acid folding and hybridization prediction. Nucleic Acids Res 31: 3406–3415. 12824337 10.1093/nar/gkg595 PMC169194

