# Supplementary material for: Characterization of microRNAs Expressed during Secondary Wall Biosynthesis in Acacia mangium
Source: PLoS One. 2012 Nov 27;7(11):e49662. doi: 10.1371/journal.pone.0049662 (PMC3507875; doi:10.1371/journal.pone.0049662)
Supplement: Figure S1 — Nucleotide sequence of the nine mRNA target of amg-miR166 obtained via 3′ and 5′ RACE. (DOC) [file pone.0049662.s001.doc]

**Full length HD-ZIP III (1)**

TTCCAAATGCCTGGAATGAAGCCTGGTCCGGATTCTGTTGGGATCTTTGCCATTTCGCGAAGTTGCAGTGGAGTGGCTGCGCGAGCATGCGGTCTTGTTAGTTTAGAACCTACAAAGATCGCAGAGATCCTAAAAGATCGTCCATCTTGGTTCCGGGATTGCCGGAGTCTTGAAGTATTCACAATGTTCCCTGCCGGTAATGGTGGGACAATTGAACTCATATACACACAGACATATGCTCCAACTACACTGGCTCCTGCCCGGGATTTCTGGACTCTTAGATACACTACAAGTTTGGACAATGGAAGTCTTGTGGTTTGTGAGAGATCACTGTCTGGTTCAGGTGCTGGCCCTAATGCAGCAGCCGCAGCTCAGTTTGTAAGAGCTGAAATGTTCCCAAGTGGCTACCTGATTCGACCATGTGAAGGTGGAGGATCGATCATTCATATTGTAGACCACTTAAACCTCGAGGCATGGAGTGTGCCAGAGGTTCGTAGACCCCTCTATGAATCATCAAAAGTGGTGGCTCAAAAAATGACAATTGCAGCACTACGCTATATTAGGCAAATCGCTCAGGAAACAAGTGGCGAAGTTGTTTATGGTTTAGGCAGACAGCCAGCTGTTCTGCGAACCTTCAGCCAAAGGCTAAGCAGAGGCTTCAATGACGCTGTCAATGGATTTAATGATGACGGCTGGTCCATATTGAATTGTGATAATGCTGAGGACGTGGTTATCGCAATCAACTCAACCAAGAATCTCAGTGCCACTTGTATTCCTGCAAGTTCCCTAACATTCCCTGGAGGAGTTATCTGTGCAAAAGCTTCCATGTTGCTCTAGAATGTTCCTCCGGCAGTCTTGGTTCGGTTTCTGAGGGAGCATCGCTCTGAGTGGGCTGAATTTGTTGATGCCTACTCTGCTGCTTCTCTTAAAGCAGGTTGCTTTGCCTATCCTGGG**ATG**AGGTCAACTAGATTCACTGGAAGTCAAATTATCATGCCTCTTGGGCATACAATTGAACATGAAGAGATGCTTGAAGTTGTTCGTCTTGAAGGCCACTCTCTAGTTCAGGAGGATTCTTTTGCTTCAAGGGACATTCATCTCTTACAGATATGTAGTGGGATTGATGAAAATGCTGTTGGGGCTTGTTCTGAGCTTGTATTTGCTTCCATCGATGAAATGTTCCCTGATGATGCTCCATTGTTGCCATCCGGTTTCCGCATCATCCCATTGGATTCAAAATCAGGTGATAAGAAGAATGCAGTATCTGTAAATCGGACCCTAGACTTGACATCTGGTCTTGATGTGGGCCCTGCAACAAATCATGCTGCAGAAGATGCATCTTCATGCCATAATACTCGGTCGGTATTGACTATTGCCTTCCAGTTTCCCTTTGAGAGCAGCTTGCAGGAAAACATTGCAATTATGGCGCGGCAGTATGTGCGCAGTGTGATTTCATCTGTGCAGAGGGTTGCCTTGGCTATATCTCCATCTGGTATAAGCCCAACTGTGGGACCAAAACTCAGTCCTGGCTCTCCTGAAGCAGTAACTCTAGCTCACTGGATATGCCAGAGTTATAGTTTCTATATGGGAGCAGAATTACTGCAATCCGATTCTCTTGTTGGCGATTTGTTGCTGAAACAACTATGGCATCATCAGGATGCCATTTTGTGTTGTTCAGTGAGGTCCATGCCTGTGTTCATCTTTGCAAACCAGGCAGGGCTTGACATGCTGGAAACAACCCTAGTGGCTCTGCAAGATATCACATTGGATAAAATATTTGACGACTCTGGACGCAAGGCATTGTGTACAGAATTCGCCAAGTTGATGCAGGAGGGGTTTACTCCAAATGCCTGGAATGAAGCCTGGTCCGTGATGGGACGGCATGTTTCATATGAACAAGCTCTTGCATGGAAAGTTCTTGCTGAAGATAACAGTGTTCATTGCTTAGCTTTCTCTTTCATAAGTTGGTCATTTGTA**TGA**ATATTCAATGCCATATTGCCATATTTATCTAATTATTGTCTTGGTTCTCTT TTTATTGAATTACTTAAAACTTATTCCATTGTAACGAGGATTGCTGTAGTCATGTAAATACAGAACTCG

TTTTATCTGTCAAAGAAAACAAGACTCGTATTTTCTCTAAAAAAAAAAAAAAAAAAAAAAAAAAAAAG

Nucleotide sequence of HD-ZIP III (1). Nucleotide sequence of HD-ZIP III (1) with amg-miR166 binding site (highlight in yellow colour) located at the 5’ untranslated region (UTR). ATG and TGA = start and stop codon. HD-ZIP III (I) shared 91% in nucleotide level and 89% in protein level to *Glycine max* HD-ZIP III (REVOLUTA) transcription factors with e-value of 0. High level of CpG island were observed after amg-miR166 gene target sites.

**Full length HD-ZIP III (2)**

CAGATGATTGGGATGAAGCCTGGTCCGGATTCCATTGGAATCGTTGCTATTTCTCATGGTTGCACTGGCGTGGGAGCACGAGCCTGTGGTCTTGTGGGTCTTGAACCCACAAGGGTTGTGGAAATCCTCAAAGATCGGCCTTCATGGTCTCGTGATTGCCGAGCTGTTGATGTTCTGAATGTGCTGCCCACAGCTAATGGTGGAACCATAGAGCTGCTTTAT**ATG**CAGCTATATGCACCAACAACATTGGCACCTGCTCGTGACTTTTGGCTGTTGCGCTACACATCTGTTTTGGAAGATGGCAGCCTTGTGGTCTGTGAGAGGTCTCTTAAAAATATTCAAAATGGTCCAAGCATGCCACCCGTGCAGCATTTTGTGAGAGCAGAGTTGCTTCCTAGTGGGTACTTGATAAGACCTTGTGAAGGGGGTGGTTCTATCATCCACATTGTCGATCACATGGATTTGGAGCCGTGGAGTGTGCCAGAAGTACTGCGTCCATTGTATGAATCATCAACAGTGATGGCTCAGAAGACAACCATGGCGGCCCTACGCCAGCTTAGACAGATATCTCATGAGGTTTCTCAGTCTAATGTCAATGGGTGGGGCAGACGACCTGCAGCTTTAAGAGCACTTAGCCAGAGATTGAGCAGGGGTTTCAATGAGGCTCTAAATGGGTTTACTGATGAAGGATGGTCAATGATTGGCAACGATGGTGTAGATGATGTTATGATCCTGGTGAATTCATCTCCTGACAAATTAATGGGTTTAAATCTCTCCTTTGCCAATGGATTTCCATCTATTAGCAATGCAGTCTTATGTGCCAAAGCATCAATGCTGTTACAGAATGTGCCTCCGGCCATTCTCCTCAGGTTTTTGCGGGAGCATAGATCAGAATGGGCAGACAACAACATGGATGCTTACTCTGCTGCCGCCATAAAAGTTGGTCCTTGCAGCCTGCCAGGATCTCGTGTTGGAAATTATGGAGGTCAAGTTATTCTTCCTCTAGCCCACACTATTGAGCATGAAGAGCTTCTGGAAGTCATTAAATTGGATGGAATTGCTCATGCTGTTGAGGACACAATAATGCCAAGAGAGCTGTTTCTTTTGCAACTCTGTAGTGGGATGGATGAGAATGCTGTTGGTACCTGTGCTGAGCTTATATTTGCTCCAATTGATGCATCCTTTGCTGATGACGCCCCCCTTTTACCTTCTGGATTTCGCATTATTCCTCTTGATTCTTGGAAGGAAGCCTCTAGCCCAAATCGAACCCTTGACCTTGCATCTTCTCTTGATGTTGGCCCTGCTGGAAATAGGGCATC**TAATGATTA**TGCTGGAAATTCTGGCTGTATGAGATCCGTGATGACAATAGCATTTGAATTTGCATGTGAAAGCCATATGCAGGAGCATGTGGCGTCCATGGCACGACAATATGTTCGCAGCATTATATCATCAGTCCAAAGGGTGGCGTTAGCTCTTTCTCCATCTCATCTAGGCTCACAGGCTGGACTGCGACCACCGCTTGGTACTCCTGAAGCCCAAACACTTGCTCGATGGATCTGCAACAGTTATAGGTCCTACTTGGGAGTGGAGCTGCTAAAGACCAATAATGAGGGGAATGAATCGATACTCAAGGCCTTGTGGCATCACTCAGATGCTCTTCTGTGCTGCACTTTAAAGGCAACGCCAGTGTTCACTTTTGCCAACCAGGCAGGGCTTGACATGCTGGAAACTACCTTAGTTGCACTGCAAGACTTGAGTTTGGAGAAAATATTTGATGATCATGGGAGAAAGATCCTCTGTTCTGAGTTTCCCCAGATTATACAACAGGGCTTTGCTTGCCTTCAAGGTGGCATCTGTGTCTCGAACATGGGGCGGCCGGTCTCACACGAGAGAGTTGTGGCTTGGAAAGTGCTGAATGAAGAGGAGAATGCTCATTGCATCTGCTTTATGTTTGTGAACTGGTCTTTTGTT**TGA**TGGTTTTATATCCCAAGAATAGTACATTAAAATGTTATAGTTGAAGTCAGTGTACCCCAACTTTTGTGAACTTGCGTCTTGTTTGGTAATTTGATGTGCTACGTATCTACTGGATTTATTTGAAAAAAAAAAAAAAAAAAAAAAAAAAAAA

Nucleotide sequence of HD-ZIP III (2). Nucleotide sequence of HD-ZIP III (2) with amg-miR166 binding site (highlight in yellow colour) located at the 5’ untranslated region (UTR). HD-ZIP III (2) contains TAATNATTA – like sequence. ATG and TGA = start and stop codon. HD-ZIP III (2) shared 92% in nucleotide and protein level to *Medicago truncatula* HD-ZIP III (ATHB15 group) transcription factors with e-value of 0. High level of CpG island were observed after amg-miR166 gene target sites.

**Full length HD-ZIP III (3)**

TCGAATGCGACTCACTATAGGGCAAGCAGTGGTATCAACGCAGAGTAC**ATG**GGTACACCTGAGCAGGTTGAAGCCCTTGAGAGGCTTTATCATGAATGTCCAAAACCCAGTTCCATTCGCCGCCAGCAGCTCATCCGTGAGTGCCCTATTCTTTCCAACATTGAACCCAAACAAATCAAAGTTTGGTTCCAGAACAGAAGGTGTAGAGAGAAGCAAAGGAAAGAGGCCTCACGACTTCAAGCTGTGAATAGGAAGCTGACGGCCATGAACAAACTTTTGATGGAGGAGAATGATAGGTTGCAGAAGCAGGTCTCTCAATTGGTGTATGAAAATGGCTACTTCCGCCAACATACTCAAAATACTACGCTTGCAACCAAAGACACAAGCTGTGAATCAGCCGTGACGAGCGGCCAACACAGTTTGACTACTCAGCATCCCCCAAGGGATGCAAGTCCTGCAGGACTCTTGTCCATTGCAGAAGAGACTTTAGCAGAGTTTCTTTCAAAGGCTACTGGGACTGCTGTTGAGTGGGTCCAAATGCCTGGAATGAAGCCTGGTCCGGATTCCATTGGAATTGTTGCTATTTCTCATGGTTGCACTGGTGTGGCAGCACGTGCCTGTGGTCTAGTGGGTCTAGAACCCACAAGAGTTGCAGAAATACTCAAGGATCGGCCGTCATGGTTTCGTGATTGCCGAGCTGTTGATGTTCTGAATGTGCTGCCCACAGCAAATGGTGGAACCATAGAGTTGCTTTATATGCAGCTATATGCCCCAACCACATTGGCTCCTGCTCGAGACTTTTGGTTGTTGCGCTATACTTCTGTTCTAGAAGATGGGAGCTTTGTGATCTGTGAGAGGTCTCTTAAAAATACTCAAAATGGTCCAAGCATGCCTCCCGTGCAGCATTTTGTGAGAGCTGAGATGCTGCCTAGTGGTTACCTAATAAGACCTTGTGAAGGGGGTGGGTCTATTATCCACATTGTTGATCACATGGATTTGGAGCCATGGAGTGTGCCAGAAGTGTTACGCCCTCTATATGAATCATCAACAGTGCTAGCTCAGAAGAAAACGATGGCGGCCTTACGACAGCTGAGACAGTTATCTCATGAGGTTTCTCAGCCCAATGTCACCGGCTGGGGCAGACGACCAGCGGCACTGCGAGCACTAAGCCAGAGACTTAGCCGGGGTTTCAATGAGGCACTCAATGGGTTTAGTGATGAGGGGTGGTCAATGATTGGCAATGATGGTATAGATGACGTTACAATCATGGTTAATTCGTCTCCTGACAAGCTAATGGGACTAGATCTTTCCTTTGCCAATGGATTTCCATCCATCAGTAATGCTGTGCTGTGTGCCAAAGCATCAATGCTATTGCAGAATGTGCCTCCGGCCATTCTCTTAAGGTCCCTGCGGGAGCACAGATCAGAATGGGCAGACAACAACATGGATGCTTACTCAGCCGCCGCCATTAAAGTTGGTCCGTGTAGCTTACCAGGATCTCGTGTTGGAAATTATGGAGGCCAAGTTATACTTCCTCTGGCTCACACCATTGAGCATGAAGAGTTTCTTGAAGTGATTAAATTGGAAGGGTTAGCACATTCTCCTGAAGATGCGATCATGCCTAGAGAGGTGTTTCTTTTGCAACTCTGTAGTGGAATGGATGAGAATGCTGTTGGCACTTGTGCTGAACTTATATTTGCTCCAATTGATGCATCCTTTGCTGATGATGCCCCACTTTTACCTTCTGGGTTCCGCATCATTCCTCTTGATTCTGGGAAGGAAGCGTCCAGTCCGAATCGCACCCTTGATCTTGCATCTGCACTTGACATTGGCCCAGCTGGGAATAAACAATC**TAATGATTA**TTCTGGAAACTCTGGCTGCATGAGATCTGTTATGACTATAGCATTTGAATTTGCATTTGAAAGTCATATGCAAGAACACGTTGCAACAATGGCACGACAGTATGTTCGTAGCATTATATCATCAGTCCAAAGGGTGGCGCTAGCGCTTTCTCCTTCGCATCTGAATTCTCTAGCTGGGCTTAGGTCACCACTGGGTACTCCTGAAGCACAGACACTTGCTCGTTGGATATGCAACAGTTATAGGTGCTACTTGGGTGTGGAGCTACTAAAGTCCAGTAATGAAGGAAATGAATCCATCCTTAAGTCCTTGTGGCATCACTCTGACGCAATTCTGTGCTGCTCCCTGAAGGCATTGCCGGTCTTCACATTTGCGAACCAGGCAGGGCTTGATATGCTGGAGACTACGTTAGTTGCACTGCAAGACATCGTTTTGGAGAAGATATTTGATGAACATGGACGTAAAATACTCTGTTCAGAGTTTCCTCAGATCATACAA**TAG**GGTTTTGCATGCCTTCAGGGAGGTATCTGTCTGTCGAGCATGGGGAGGCCGGTGTCGCACGAGAGAGTTGTGGCTTGGAAGGTGCTGAATGAAGAAGAGAATGCTCATTGTATCTGTTTTATGTTTATGAACTGGTCCTTTGTTTGAAGTTTGATGTCTAAATAGACTGAGGATTGAACCAGTGTTGAACATGAATCATTCTGTATTCTCAAGACCTTGTGTTTTGTTGGTTGTTCTTCTGTATTTATCTTCCGCCAAAGACCTGGATTGCATATGAAGACAATATGTTATATTAAAAAAAAAAAAAAAAAAAAAAAAAAAA

Nucleotide sequence of HD-ZIP III (3). Nucleotide sequence of HD-ZIP III (3) with amg-miR166 binding site (highlight in yellow colour) located at the 5’ untranslated region (UTR). HD-ZIP III (3) contains TAATNATTA – like sequence. ATG and TAG = start and stop codon. HD-ZIP III (3) shared 91% in nucleotide and 92% in protein level to *Medicago truncatula* HD-ZIP III (ATHB15 group) with e-value of 0. High level of CpG island were observed after amg-miR166 gene target sites.

**Full length HD-ZIP III (4)**

TCGATAAGCAGTGGTATCAACGCAGAGTACATGGGGATGTCTGATTGATCTCCTCCTGACTGAAACCCAGCGTATAGTTAGACGGG**ATG**ATGGCGGTGAGCTCGGCGTGCAAAGATGGGAGCAAGGTGGCGATGGATAACGGCAAGTACGTGCGTTACACACCGGAGCAGGTGGAGGCTCTGGAGAGGCTCTATCACGAATGCCCCAAACCCAGTTCCATGCGCCGTCAGCAGATTATCCGAGAGTGTCCCATTCTCTCAAACATCGAGCCCAAACAGATCAAGGTTTGGTTTCAGAATCGAAGATGTAGAGAGAAGCAGCGAAAAGAAGCCTCACGCCTGCAAGGTGTAAACAGAAAACTGACGGCTATGAATAAACTGCTGATGGAGGAAAACGACAGATTGCAGAAACAGGTGTCCCAGCTGGTTTACGAGAACAGCTTTTTCCGCCAACATCAGAATACGACTCTGGCAACGACGGACACAAGTTGCGAGTCGGTGGTGACGAGCGGTCAGCACCATGTGACACCTCAGCATCCACCAAGGGATGCAAGCCCAGCTGGACTTTTGTCCATTGCAGAGGAGACTCTTGCAGAGTTTCTTTCAAAGGCCACTGGAACTGCCGTGGAGTGGGTCCAGATGATTGGGATGAAGCCTGGTCCGGATTCCATTGGAATCGTTGCTATTTCTCATGGTTGCCCTGGAGTGGCAGCCCGCGCTTGCGGCCTTGTGGGTTTGGAACCTACGAGGGTTGCCGAAATCCTCAAAGATCGGCCCTCATGGTGTCGTGACTGCCGAGCTGTGGATGTTCTAAATGCGATGACCACAGGAAATGGGGGAACCGTTGAACTGCTTTACATGCAGTTGTATGCCCCAACAACTTTGGCTCCAGCTCGTGACTTCTGGCTGCTGCGTTACACTTCTGTTTTGGAGGATGGTAGTCTTGTGGTCTGTGAAAGATCACTTAACAACACTCAGAATGGTCCTACTATGCCACCAGCGCAGCATTTTGTGAGAGCAGACATGCTCCCAAGCGGGTATCTGATTAGACCTTGTGAAGGAGGTGGATCCATCATTCATATTGTTGATCACCTCAACTTAGAGCCTTGGAGTGTACCTGAAGTGTTACGCCCACTCTACGAGTCTTCAACGTTGTTTGCTCAAAAGACAGCCATGGCGGCTTTACGTCGCCTGAGGCAAATCTCTCAAGAAGTTTCCCAGCCAAATGTCACCGGATGGGGAAGAAGGCCTGCAGCTTTGCGAGCACTGAGTCAGCGTTTGAGCAGGGGCTTCAACGAAGCTGTCAATGGATTTGCAGACGATGGCTGGTCCATGTTGGAAAGTGATGGCATTGATGATGTCACCCTTCTTGTGAATTTATCTCCTAACAAGACCATGGGAGTAAATATTGGTTATAACAATGGGTTTCTTTCGATGAGCAGTTCCGTGCTATGTGCAAAAGCGTCCATGTTGTTACAGAATGTCCCTCCTGCAATTCTTCTTAGATTCTTGCGGGAGCATCGATCAGAGTGGGCAGATAGCAATATTGATGCTTACTCAGCCGCTGCCATCAAAGCTGGTCCCTGTGCCTTGCCTGGGGTTCGAGCAGGAGGATTTGGTGGTCAGGTTATTCTTCCACTGGCGCACACAATTGAGAATGAAGAGTTTATGGAGGTCATCAAGCTTGAAAATATGGGATACTATAGAGAAGACATGACTATTCCGGGCGATATTTTCCTCTTGCAACTTTGTAGTGGAGTAGATGAGCGTGCTGTTGGCAGTTGTGCAGAACTCATTTTTGCTCCCATTGATGCATCTTTCTCTGATGATGCTCCCATTTTACCTTCTGGTTTTCGCATCATACCTCTTGATTCAGGCATGGATGCTGCTACTCCAAACCGAACACTTGATCTTGCTTCTGCTCTTGATGTTGGAACCACTGGAAACAAATCAGCTGGCGATAATGTTGCTCAATCTGGGAGCACAAAATCTGTGATGACAATAGCATTTCAGTTTGCATTTGAAGTCCATCTTCAAGATAGTGTAGCAGCTATGGCTAGGCAGTATGTTCGGAGTATTATTGCATCTGTTCAAAGGGTAGCATTGGCACTATCCCCTTCCCACTTTGGTTCGCAAAACGGTCTTCGCCCTCCACCTGGCACTCCTGAGGCACAAACACTTGCTCAGTGGATCTGTCATAGCTATAGGAGCTATCTAGGGGTAGAACTACTAAAATGTGAAGGCAGTGAATCCATTCTCAATTCCCTTTGGCATCACTCTGATGCAGTCTTGTGCTGCTCTTTAAAGGCTTTGCCGGTTTTTACATTTGCAAATCAAGCGGGACTTGACATGCTTGAGACAACTTTGGTAGCTCTTCAAGACATCACTCTCGATAAAATTTTTGACGAGAATGGGAAGAAAACCCTGTTTTTTGAGTTGCCCCAGATAATGCAGCAGGGCTTTATGTGTATGCAAGGAGGTATCTGCTTGTCCAGCATGGGGAGGCCAGTGTCTTACGAAAGAGCGGTTGCATGGAAAGTAATGAACGAAGAAGAAACGGCCCATTGCATTTGTTTCATGTTCATCAACTGGTCTTTTGTC**TGA**TAGGTGAATAGTATATATACAGAATCCAGGAAAGTAGAGCAGAACAGAACAGTATCATCCTCTTTTAATTTTTTTTTTTAATTTTGGAGAATCCCTAGATTGCTATATATATAATTTACTCTTGTGGGCATGCTGCTCCAAAAAGAAAAAAAAAAAAAAAAAAAAAAAAAAAA

Nucleotide sequence of HD-ZIP III (4). Nucleotide sequence of HD-ZIP III (4) with amg-miR166 binding site (highlight in yellow colour) located at the 5’ untranslated region (UTR). ATG and TGA = start and stop codon. HD-ZIP III (4) shared 89% in nucleotide and 88% in protein level to *Populus trichocarpa* class HD-ZIP III (ATHB8 group) with e-value of 0. High level of CpG island were observed after amg-miR166 gene target sites.

**HD-ZIP III (5)**

TTCTCGGGAAAAAAGTTTTTGCCCCGGTTTTCCCCACGCTTTTTGGGTGGGCCCTTTGGGAATACTCCAAGTTTTGCTTTCCCACCGGGGTGGGGGGTTTCTCCCCATGGTGGCCCCGGGGGGGGGGGGGAATTTCCCGGGGGTTTTTAATAGGCCCCCTTTAGGGCAAACCAGGGTTTTCACGCGGGGGATTTTTTTTTTTTTTTTTTTTTTTTTTTTTTTGCAATATAATGAAACGAATATCATTGACGACAGATTCTTCTTGGCACAAATCTTCTTTGCTGGCATACTTTTCTTAGCGCGCAAGAACAAGCTTGGAACGTAATATTTATATACACATTCGACCAAAACGTACGAATACTAATATACATAAAAATTAAACAACGACGACGCTGAGATTATTTGACGGTACTGTTATTCACGTCATCTAGCCTCTAGATCACGAAGTGTTTGCTGATTGCATTGTTAAGTGGCAATGAACACGGACATGCGCCTTTCATCTTCATCTTGATATATAATTGCTTAAAATAATAACAAAAAGTAACCTATAATTTAGACATGAAAATATATTCCTTTAAAATAAAGAGAGAGACATAGCCAAGTGCCTTGTAGCGCGATGACATTAAAGCCAGCCTTAGGAGCTGGCTTTCCTCTTTGTCCAGGGTTCGAGCGCCCTGCCTCCCCCTCTCCCTACTGGCTGTATCAATAAAAAAAGAAAAAAGAGAGAGATATAGCTATGAAGTGTCATTAAATGGATATGGGAGGCCCTTTATTAAAAAAAAATGAGGGAAGTTAAAAGTAGGAAAGAGTGACAAGCATGGTTGATAGTGGAATGGAGAGGAACATGAATGGGGATGTATTAGAAGAAGAGGAGGAGGGTGGAATGAAGCCTGGTCCGAGA

**HD-ZIP III (6)**

TTTTTTTTTTTTTCGACCAAGCTCATGGATTATGAACAAAAAGAATGCGGCGTAAAAAATGGTATATATAACCATGTCCCAACTCTTATATTTTGCATGCATATTGTGCATATGCTCACAGATACAAGGAGAAAGATACAGAGAATCAATTTGCAGTTTCACCAGCAGGGCCAGGGCGTGCATTCAACAAAGGTTGAAGAGCTTTGACTACAATGCTCATGTTTGGTCTGAAGTCAGCCTCATATTGCACGCATAGCGCAGCAACAGCAGCCATCTTAGCAACAGCTTTGGGTGGGTAATCTCCTCCTAGTTTTGTATCAACACACTGCCTAACTTTATCTTCGCTGAGTTTTGGTGTAGCCCAAGTAACCAGACTCTGCTGTCCACGAGGTAGTGTATGATCAACAGGCTTCCTTCCAGTCAAAAGTTCCAGAAGGACAACACCAAAACTGTACACGTCACTCTTAGCATTCAGTTGTCCGGTCATTGCATATTCTGGTGCATGATAGCCAAAGGTTCCAAGGACACGAGTGGAATGAAGCCTGGTCCGAGA

**HD-ZIP III (7)** TTCCAGGGAAACGCTTTGCCCATGATTACGCCCAAGGTATTTTAGGGGGCCCTTTAGAATAATCCAAGGTTTGGCATCCCAACGGGGTGGGGGGGTTTCCCCAAAATGGTGGACCCTGCAGGGGGGGCGGGGAATTCCCTTAGGGGTTTTTTATACGGCCCCCCTTTAGGGGCAGCAGGGGGTTTAAAGCAGAGGAATTTTTTTTTTTTTTTTTTTTTTTTTTTTTGGAGAGCGAACGAATAGAACAAGTGGGAAGAATATTCAGATTTTGTTACAAAATTCATATCCAAGGCTTCATCTCCCTCCTCATACTCTGAAATCTGAACAGCTAAATCAACTTACAAGGGGATCAGACATGAGTGTTCTTCACTGGAGCAACAACTATATATATAAAATTGTTGTAAAAGTAACTGAGTCTCATATTTTGCTAACTATCCAGATTATATAAAGACCAATCCCTGCATTCTAAATTACGGCTTTCAATTCCAATCTAGTGGGAGGAAAAAAATCTCAGGGCTTCCACTCAATACATTGAAAGAATACATCCTTCCTCATGCTCAAAACCCTGTGATTTTACAGCCAACACTGTTTCATTTTACCATCATCAACCAGAGGCCTCCACTAGAAAACTTGCTCTCTCCAGCAACAAAACTAGGGAAGTTCTCAGGTCTAAACTCACAGATAAGCCCTAGTCGCACTTAATGTTTCGGTATATCCTTCTCACAAACAAATTTGAACCAAGATAACCCACAGCCCCGCAGAGAATTCCAAGTCCAAGACAAAACATCAAGGTGTATCCAAAATAGAAGCTAGTTTGGAATGAAGCCTGGTCCGAGA

**HD-ZIP III (8)**

GCCCCACAGAAGACCTTTTCCCCCGTTTTTCCCCACTTTTTTGGGGGCCCCTTTTAAAAACCCCCATTTTGTTTCCCAACGGGGGGGGGGTTTCCCCCATGGGGGCCCCCCGGGGGGGGGGGAATTTTCTGGGGGTTTTTTAAGGCCCCCCTTTTGGGGAACCCGGGGGTTTTACCCGGGGAAATTTTTTTTTTTTTTTTTTTTTTTTTTTTTGGGTTTTTTTTTTTTTTTTTTTTTTTTTTTTTTTTTTTCTCATGAATAGCACAACGCTAATAGATATCCATAGAGAAACAAAATATTCCAAGTGTTTTGAACATCCGAAAAAGAAAAGGTACATAGTAAAATGAAGACAGCATTGACGCTATGAATCCTATTTTTGAACCTAAAACAGAGAAGTTCCCTTTCCCTTCTTATCTCCTCCAATCTCAAAGTGCTTGCACCTCTTAATAGCATGTTGGGATACATGTTTGCAACTCTGGCATTGCAGCCTCAAGACAATCTTCTTGGTGGTTTTAGCCTTTTTGTGGAACACAGGCTTTGTCTGTCCACCGTATCCTGACTGCTTACGGTCATAACGGCGCTTTCCCTGAGCTGCAAGGCTATCCTTGCCTTTCTTGTATTGAGTAACCTTGTGCAAGGTGTGCTTCTTGCACTCCTTATTCTTGCAGTAAGTCTTCTTGGTCTTTGGAACGTTCACCATGATGTGGATCAACCTGAGCTCCGCGCCGCTGAAGAGTGAAGAAGACGAGCTCGCTGAGGAATGAAGCCTGGTCCGAGA

**HD-ZIP III (9)**

ACCCCCCAGGGGAGTTTCCAGGGGAAACGCTTGGTTTCTTTAAAGCCCGTCGGGTTTCGCCACCTTTGACTTGAGGGTCGATTTTTTGGATGCTTGTCAGGGGGGCGGAGCCTATGGAAAAACGCCAGCAACGGGGCCTTTTTACGGTTCCTGGCCTTTTGCTGGCCTTTTGCTCACATGTTCTTTCCTGCGTTATCCCCTGATTCTGTGGATAACCGTATTACCGCCTTTGAGTGAGCTGATACCGCTCGCCGCAGCCGAACGACCGAGCGCAGCGAGTCAGTGAGCGAGGAAGCGGAAGAGCGCCCAATACGCAAACCGCTTCTCCCCGCGCGTTGGCCGATTCATTAATGCAGCTGGCACGACAGGTTTCCCGACTGGAAAGCGGGCAGTGAGCGCAACGCAATTAATGTGAGTTAGCTCACTCATTAGGCACCCCAGGCTTTACACTTTATGCTTCCGGCTCGTATGTTGTGTGGAATTGTGAGCGGATAACAATTTCACACAGGAAACAGCTATGACCATGATTACGCCAAGCTATTTAGGTGACACTATAGAATACTCAAGCTATGCATCCAACGCGTTGGGAGCTCTCCCATATGGTCGACCTGCAGGCGGCCGCGAATTCACTAGTGATTCCAAATGCCTGGAATGAAGCCTGGTCCGAGA

Figure S1: Nucleotide sequence of the nine mRNA target of amg-miR166 obtained via 3’ and 5’ RACE. HD-ZIP III (1-4) obtained via 5’ and 3’RACE mapping of amg-miR166 cleavage site while HD-ZIP III (5-9) obtained via 5’RACE mapping of amg-miR166 cleavage site. -amg-miR166 binding site are highlighted in yellow. Nucleotide and protein similarity of HD-ZIP III (1-4) obtained via blast analysis are shown. Blast analysis at nucleotide and protein level indicated HD-ZIP III (5-9) are novel gene targets of amg-miR166 (Blast output not shown).
