# Supplementary material for: Characterization of microRNAs Expressed during Secondary Wall Biosynthesis in Acacia mangium
Source: PLoS One. 2012 Nov 27;7(11):e49662. doi: 10.1371/journal.pone.0049662 (PMC3507875; doi:10.1371/journal.pone.0049662)
Supplement: Figure S2 — Binding energy characterization between amg-miRNA members with various mRNA isoforms from Arabidopsis . (DOC) [file pone.0049662.s002.doc]

**a. amg-miR159 - MYB**

**1. *Arabidopsis thaliana* myb domain protein 65 (MYB65) mRNA, complete cds**

miRNA 21 AUCUCGAGGGAAGUUAGGUUU 1

:.:::::::::::: :::::

Target 1155 UGGAGCUCCCUUCAUUCCAAU 1175

**2. *Arabidopsis thaliana* myb domain protein 33 (MYB33) mRNA, complete cds**

miRNA 21 AUCUCGAGGGAAGUUAGGUUU 1

:.:::::::::::: :::::

Target 1249 UGGAGCUCCCUUCAUUCCAAU 1269

**3. *Arabidopsis thaliana* putative transcription factor MYB120 (MYB120) mRNA, complete cds**

miRNA 20 UCUCGAGGGAAGUUAGGUUU 1

:::::::::::: :::::

Target 1170 GCAGCUCCCUUCAAACCAAA 1189

**4. *Arabidopsis thaliana* DNA-binding protein (MYB104) gene, complete cds**

miRNA 21 AUCUCGAGGGAAGUUAGGUUU 1

:.:::::::::::: :::::.

Target 848 UGGAGCUCCCUUCAUUCCAAG 868

**5. *Arabidopsis thaliana* myb domain protein 101 (MYB101) mRNA, complete cds**

miRNA 21 AUCUCGAGGGAAGUUAGGUUU 1

:::::::.::::::: :::::

Target 1004 UAGAGCUUCCUUCAAACCAAA 1024

**6. *Arabidopsis thaliana* MYB transcription factor (At5g06100) mRNA, complete cds**

miRNA 21 AUCUCGAGGGAAGUUAGGUUU 1

:.:::::::::::: :::::

Target 938 UGGAGCUCCCUUCAUUCCAAU 958

**b. amg-miR156 - squamosa promoter-binding-like protein**

**1. *Arabidopsis thaliana* squamosa promoter-binding-like protein 15 (SPL15) mRNA, complete cds**

miRNA 21 CACGAGAGAUAGAAGACAGUU 1

::::::::: :::::::::::

Target 937 GUGCUCUCUCUCUUCUGUCAA 957

**2. *Arabidopsis thaliana* squamosa promoter-binding-like protein 2 (SPL2) mRNA, complete cds**

miRNA 21 CACGAGAGAUAGAAGACAGUU 1

::::::::: :::::::::::

Target 1446 GUGCUCUCUCUCUUCUGUCAA 1466

**3. *Arabidopsis thaliana* squamosa promoter-binding-like protein 11 (SPL11) mRNA, complete cds**

miRNA 21 CACGAGAGAUAGAAGACAGUU 1

::::::::: :::::::::::

Target 1253 GUGCUCUCUCUCUUCUGUCAA 1273

**4. *Arabidopsis thaliana* squamosa promoter-binding-like protein 10 (SPL10) mRNA, complete cds**

miRNA 21 CACGAGAGAUAGAAGACAGUU 1

::::::::: :::::::::::

Target 1188 GUGCUCUCUCUCUUCUGUCAA 1208

**5. *Arabidopsis thaliana* squamosa promoter-binding-like protein 9 (SPL9) mRNA, complete cds**

miRNA 21 CACGAGAGAUAGAAGACAGUU 1

::::::::: :::::::::::

Target 845 GUGCUCUCUCUCUUCUGUCAA 865

**c. amg-miR172 - APETALA 2**

**1. *Arabidopsis thaliana* Floral homeotic protein APETALA 2 (AP2) mRNA, complete cds**

miRNA 21 GACGUCGUAGUAGUUCUAAGA 1

::::::::::::::.::::::

Target 1329 CUGCAGCAUCAUCAGGAUUCU 1349

**2. *Arabidopsis thaliana* AP2-like ethylene-responsive transcription factor TOE2 (TOE2) mRNA, complete cds**

miRNA 20 ACGUCGUAGUAGUUCUAAGA 1

:::::::::::::.::::::

Target 1811 UGCAGCAUCAUCAGGAUUCU 1830

**3. *Arabidopsis lyrata* subsp. lyrata hypothetical protein, mRNA**

miRNA 21 GACGUCGUAGUAGUUCUAAGA 1

::::::::::::::.::::::

Target 1320 CUGCAGCAUCAUCAGGAUUCU 1340

**d. amg-miR394 - F-box**

**1. *Arabidopsis thaliana* F-box only protein 6 (AT1G27340) mRNA, complete cds**

miRNA 20 CCUCCACCUGUCUUACGGUU 1

:::::: :::::::::::::

Target 1373 GGAGGUUGACAGAAUGCCAA 1392

**e. amg-miR168 - AGO1**

**2. *Arabidopsis thaliana* protein argonaute (AGO1) mRNA, complete cds**

miRNA 21 AAGGGCUGGACGUGGUUCGCU 1

::::::: :::::.::::: :

Target 521 UUCCCGAGCUGCAUCAAGCUA 541

**Figure S2.** Gene target characterization between amg-miRNA members with various mRNA isoforms from *Arabidopsis*.

Characterization of the miRNA: mRNA cleavage site using psRNA Target Prediction Server. Various different mRNA sequence were download from NCBI and was used to search for the energy binding with amg-miRNA members (amg-miR172, amg-miR168, amg-miR394, amg-miR156 and amg-miR159). All the various mRNA as presented have antagonistic roles although they belong to same miRNA family.
