# Supplementary material for: Characterization of microRNAs Expressed during Secondary Wall Biosynthesis in Acacia mangium
Source: PLoS One. 2012 Nov 27;7(11):e49662. doi: 10.1371/journal.pone.0049662 (PMC3507875; doi:10.1371/journal.pone.0049662)
Supplement: Figure S3 — Semi-quantitative reverse transcription PCR analysis of 12 highly conserved miRNA families in Leaf (L), Phloem (P), Xylem (X), Compression Wood (CW) and Tension Wood (TW). (DOC) [file pone.0049662.s003.doc]

**1. amg-miR394**

L P X CW TW


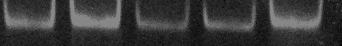
 amg-miR394 (NSW19)


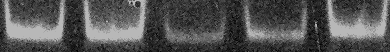
 amg-miR394 (NSW20)


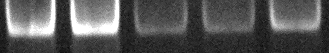
 amg-miR394 (ERC22)


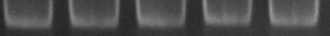
control

**2. amg-miR166**

L P X CW TW


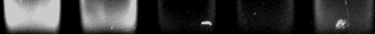
 amg-miR166 (NSW19)


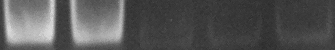
 amg–miR166 (NSW20)


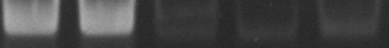
 amg-miR166 (ERC22)


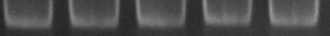
control

**3. amg-miR168**

L P X CW TW


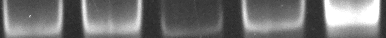
 amg-miR168 (NSW19)


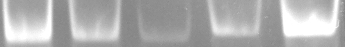
 amg-miR168 (NSW20)


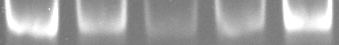
 amg-miR168 (ERC22)


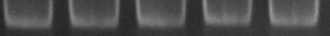
control

**4. amg-miR156**

L P X CW TW


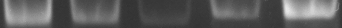
 amg-miR156 (NSW19)


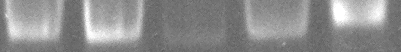
 amg-miR156 (NSW20)


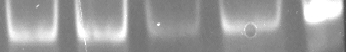
 amg-miR156 (ERC22)


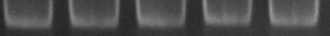
 control

**5. amg-miR159**

L P X CW TW


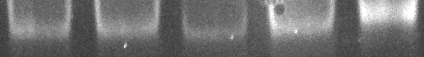
amg-miR159 (NSW19)


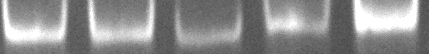
amg-miR159 (NSW20)


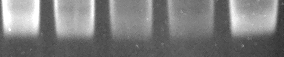
 amg-miR159 (ERC22)


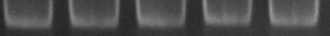
control

**6. amg-miR172**

L P X CW TW


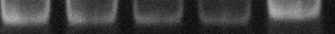
 amg-miR172 (NSW19)


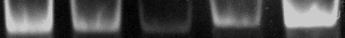
amg-miR172 (NSW20)


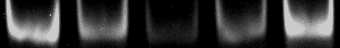
 amg-miR172 (ERC22)


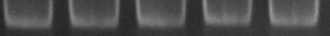
control

**7. amg-miR164**

L P X CW TW


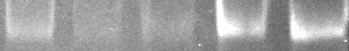
 amg-miR164 (NSW19)


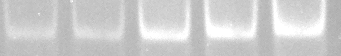
 amg-miR164 (NSW20)


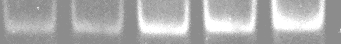
 amg-miR164 (ERC22)


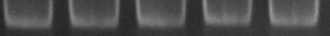
control

**8. amg-miR162**

L P X CW TW


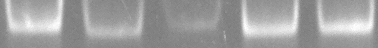
 amg-miR162 (NSW19)


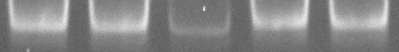
 amg-miR160 (NSW20)


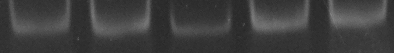
 amg-miR162 (ERC22)


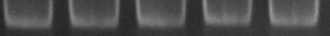
 control

**9. amg-miR396**

L P X CW TW


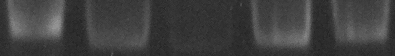
 amg-miR396 (NSW19)


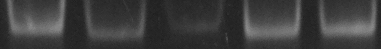
 amg-miR396 (NSW20)


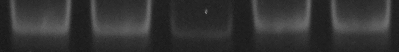
 amg-miR396 (ERC22)


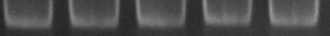
 control

Figure S3 Semi-quantitative reverse transcription PCR analysis of twelve highly conserved amg-miRNA families in Leaf (L), Phloem (P), Xylem (X), Compression Wood (CW) and Tension Wood (TW) in three different *A. mangium* trees (NSW19, NSW20 and ERC22) of 4 years old. 5.8 S ribosomal RNA was used to normalize cDNA amounts from various tissues using RT-qPCR. Each analysis was done in triplicate. Control shown above indicates that all the various tissues have same concentration (concentration was checked using RT-qPCR).

From the 12 highly conserved amg-miRNA families, three amg-miRNA families (amg-miR160, amg-miR167 and amg-miR403) were unable to be detected using semi-quantitative reverse transcription PCR across various tissues. Validation using quantitative real time PCR indicated that these three amg-miRNA families have very high ct value which indicate their low abundance. amg-miR164, amg-miR162 and amg-miR396 showed no obvious differences in intensity between compression wood and tension wood.
