# Supplementary material for: Characterization of microRNAs Expressed during Secondary Wall Biosynthesis in Acacia mangium
Source: PLoS One. 2012 Nov 27;7(11):e49662. doi: 10.1371/journal.pone.0049662 (PMC3507875; doi:10.1371/journal.pone.0049662)
Supplement: Table S1 — The 6 highly conserved plant miRNA families with strong differences in the expression level in each of the isoforms between low lignin Am54 and high lignin Am48. (DOC) [file pone.0049662.s004.doc]

**Table S1.** The 6 highly conserved plant miRNA families with strong differences in the expression level in each of the isoforms between low lignin Am54 and high lignin Am48 (Ong and Wickneswari, 2011)

miRNA Family Sequence ID miRNA Sequences Counts Counts Target

(5’ 3’) Am54 Am48

amg-miR156 3153(21) UUGACAGAAGAGAGUGAGCAC 130 22 Squamosa promoter

82(21) UUGACAGAAGAUAGAGAGCAC 3605 1001 Binding Protein

669(20) UGACAGAAGAUAGAGAGCAC 48 17 (SBP) box

23(20) UGACAGAAGAGAGUGAGCAC 1557 519

amg-miR159 966(20) UUGGAUUGAAGGGAGCUCUA 97 11 MYB transcription

87(21) UUUGGAUUGAAGGGAGCUCUA 2967 958 factor

214(19) UUUGGAUUGAAGGGAGCUC 94 39

amg-miR166 256(21) UCUCGGACCAGGCUUCAUUCC 984 324

2731(21) UUUCGGACCAGGCUUCAUUCC 79 26 HD-Zip transcription

2556(21) CUCGGACCAGGCUUCAUUCCU 86 28 factor

933(20) UCUCGGACCAGGCUUCAUUC 22 11

1090(21) UCGGACCAGGCUUCAUUCCCC 407 75

amg-miR168 1254(21) UCGCUUGGUGCAGGUCGGGAC 88 64

2219(21) UCUCUUGGUGCAGGUCGGGAA 72 34

2015(21) UCGCUUGGUUCAGGUCGGGAA 46 37

1955(21) UCGCUUGGUGCAGGUCGGGCA 81 39

567(21) UCGCUUGGUGCAGGUCGGGAU 394 139 Argonaute (AGO1)

623(21) UCGCUUGGUGCAGGGCGGGAA 75 128

2988(21) UCGCUUGGUGCAGUUCGGGAA 67 23

2482(21) CGCUUGGUGCAGGUCGGGAAU 70 29

3375(21) UCGCUUGGUGCAUGUCGGGAA 58 21

3691(21) AUCGCUUGGUGCAGGUCGGGA 20 19

3(20) GUGGCAUGUGUGGAACGGCA 2974 1723

4(20) CGCUUGGUGCAGGUCGGGAA 2841 1053

1418(21) CGCUUGGUGCAGGUCGGGAAC 210 56

2219(21) UCUCUUGGUGCAGGUCGGGAA 72 34

3749(21) UCGCUUGGUGCAGGUCGGCAA 33 18

3953(21) UCGCUUGGUGCAGGACGGGAA 45 17

3965(21) UCGCUUGGUGCAGGUCCGGAA 30 17

4077(21) UCGCUUGGAGCAGGUCGGGAA 24 17

4675(21) UCGCUUGGUGCCGGUCGGGAA 21 14

4(21) UCGCUUGGUGCAGGUCGGGAA 71256 29703

amg-miR172 3681(21) GGAAUCUUGAUGAUGCUGCAU 34 19

1788(21) GUAGCAUCAUCAAGAUUCACA 73 43

1335(21) GGAAUCUUGAUGAUGCUGCAC 196 60

2849(21) AGAAUCUUGAUGAUGUUGCAG 42 25

2372(21) AGAAUCUUGAUGAUGCUGCCU 94 31

2316(21) AGAAUUUUGAUGAUGCUGCAU 73 32

3482(21) AGAAUAUUGAUGAUGCUGCAU 15 20

3499(21) AGCAUCUUGAUGAUGCUGCAU 25 20

3806(21) AGAAUCUUGAUGAUGUUGCAU 74 18

3529(21) AGAAUCUUGAUGAUGCUGCCG 52 20 APETALA2-like

598(20) GUAGCAUCAUCAAGAUUCAC 47 18 transcription factor

227(20) GAAUCUUGAUGAUGCUGCAU 75 53

226(19) GGAGCAUCAUCAAGAUUCA 18 36

82(20) AGAAUCUUGAUGAUGCUGCA 285 160

970(21) AGAAUCUUGAUGAUGCUGCAC 99 83

3108(21) AGAAUCUUGAUGAUGCUGGAG 29 22

4816(21) AGAAUCUUGAUGAUGCUGUAU 43 14

5757(21) AGAAUCUUGAUGAUUCUGCAU 25 10

6043(21) AGAAUCUUGAUGAUGAUGCAU 12 10

651(22) AGAAUCUUGAUGAUGCUGCAGU 77 38

1008(22) GGAGCAUCAUCAAGAUUCACAU 26 24

1719(22) GAAUCUUGAUGAUGCUGCAUU 17 13

620(23) AGAAUCUUGAUGAUGCUGCAGUA 48 33

10(21) AGAAUCUUGAUGAUGCUGCAG 14505 8703

11(21) AGAAUCUUGAUGAUGCUGCAU 16400 8647

amg-miR394 1391(21) UUUGGCAUUCUGUCCACCUCC 522 57

622(21) UUGGCAUUCUGUCCACCUCCC 1157 129 F-box proteins

297(20) UUGGCAUUCUGUCCACCUCC 358 40
