# Supplementary material for: Characterization of microRNAs Expressed during Secondary Wall Biosynthesis in Acacia mangium
Source: PLoS One. 2012 Nov 27;7(11):e49662. doi: 10.1371/journal.pone.0049662 (PMC3507875; doi:10.1371/journal.pone.0049662)
Supplement: Table S2 — Primers used for 5′ and 3′ mapping of the amg-miR166 cleavage sites. (DOC) [file pone.0049662.s005.doc]

**Table S2**

1. **Primers used for 5’ mapping of the amg-miR166 cleavage sites**

Target gene Inner primer (5’ RACE) Outer primer (5’ RACE)

HD-ZIP III (1) TCATCCCAGGATAGGCAAAG CAGCAATCCTCGTTACAATGG

HD-ZIP III (2) GACGCAGTACTTCTGGCACA CCAAACAAGACGCAAGTTCA

HD-ZIP III (3) AATAGACCCACCCCCTTCAC CAGGTCTTTGGCGGAAGATA

HD-ZIP III (4) CTGCTCAATCCCTGGCTAAG CAGCATGCCCACAAGAGTAA

HD-ZIP III (5) TCTCGGACCAGGCTTCATTCC CAGCAATCCTCGTTACAATGG

HD-ZIP III (6) TCTCGGACCAGGCTTCATTCC CAGGTCTTTGGCGGAAGATA

HD-ZIP III (7) TCTCGGACCAGGCTTCATTCC CAGCATGCCCACAAGAGTAA

HD-ZIP III (8) TCTCGGACCAGGCTTCATTCC CCAAACAAGACGCAAGTTCA

HD-ZIP III (9) TCTCGGACCAGGCTTCATTCC CCAAACAAGACGCAAGTTCA

1. **Primers used for 3’ mapping of the amg-miR166 cleavage sites**

HD-ZIP Forward (5’ 3’)

HB5 CCAAATGCCTGGAATGAAGCCTGGTCCG

HB9 CAGATGATTGGGATGAAGCCTGGTCCG

1. **Forward and reverse primers of the four different full lengths HD-ZIP III**

HD-ZIP III Forward (5’ 3’) Reverse (5’ 3’)

HD-ZIP III (1) CCAAATGCCTGGAATGAAGC CAGCAATCCTCGTTACAATGG

HD-ZIP III (2) CAGATGATTGGGATGAAGCC CCAAACAAGACGCAAGTTCA

HD-ZIP III (3) CAGGTTGAAGCCCTTGAGAG CAGGTCTTTGGCGGAAGATA

HD-ZIP III (4) TAGTTAGACGGGATGATGGC CAGCATGCCCACAAGAGTAA
