# Supplementary material for: Characterization of microRNAs Expressed during Secondary Wall Biosynthesis in Acacia mangium
Source: PLoS One. 2012 Nov 27;7(11):e49662. doi: 10.1371/journal.pone.0049662 (PMC3507875; doi:10.1371/journal.pone.0049662)
Supplement: Table S3 — List of the forward and reverse primers used in RT-qPCR of various HD-ZIP III and three key lignin genes in leaf, phloem, compression wood and tension wood. (DOC) [file pone.0049662.s006.doc]

**Table S3**

List of the forward and reverse primers used in RT-qPCR of various HD-ZIP III and gene targets in leaf, phloem, compression wood and tension wood**.**

HD-ZIP Forward (5’ 3’) Reverse (5’ 3’)

HD-ZIP III (1) CGCAATCAACTCAACCAAGA TCATCCCAGGATAGGCAAAG

HD-ZIP III (2) TCCTGCTCGAGACTTTTGGT AATAGACCCACCCCCTTCAC

HD-ZIP III (3) CGCCCACTCTACGAGTCTTC TCAATGCCATCACTTTCCAA

HD-ZIP III (4) CAGCCTTGTGGTCTGTGAGA GACGCAGTACTTCTGGCACA

*CCoAOMT* TTGAGAGAGATAACAGCAAAGCA GGTGGCGAGCAAGGAGTAG

*C4H* CATTGCTGGTCCCACACAT CCTGAAGTCGTTCCCGTTA

*CAD* TCATAGGGAGCATAAAGGAGACAGA TTGAAAAGGGAAAACAGAGCACA

Actin GGTAACATTGTCCTCTCTGG CATCGTATTCTGCCTTCG
