# Supplementary material for: Characterization of microRNAs Expressed during Secondary Wall Biosynthesis in Acacia mangium
Source: PLoS One. 2012 Nov 27;7(11):e49662. doi: 10.1371/journal.pone.0049662 (PMC3507875; doi:10.1371/journal.pone.0049662)
Supplement: Table S4 — List of the forward and reverse primers used in amg-miR166 precursors structure characterization. (DOC) [file pone.0049662.s007.doc]

**Table S4**

List of the forward and reverse primers used in amg-miR166 precursors structure characterization**.**

Forward (5’ 3’) Reverse (5’ 3’)

TTGAGGGGAATGCTGTCTGGTTC GGAATGAAGCCTGGTCCGAAA

TTTCGGACCAGGCTTCATTCC AGGAATGAAGCCTGGTCCGAG

TTGAGGGGAATGTTGTCTGGCTC GAATGAAGCCTGGTCCGAGA

TGTCTTTTGAGGGGAATGTTG GGAATGAAGCCTGGTCCGAGA

TCTTTTTTGAGGGGAATGTTG GGGGAATGAAGCCTGGTCCGA

TCTCGGACCAGGCTTCATTC

TTTCGGACCAGGCTTCATTCC
TCGGACCAGGCTTCATTCCCC

CTCGGACCAGGCTTCATTCCT

TCTCGGACCAGGCTTCATTCC
